# Use of polypyrrole ferrite microparticles and liquid chromatography-mass spectrometry for testing natural grass contamination by multiclass mycotoxins

**DOI:** 10.1007/s00604-023-05763-6

**Published:** 2023-04-06

**Authors:** María García-Nicolás, Natalia Arroyo-Manzanares, Natalia Campillo, Carolina Reyes-Palomo, Santos Sanz-Fernández, José Fenoll, Vicente Rodríguez-Estévez, Pilar Viñas

**Affiliations:** 1 Department of Analytical Chemistry, Faculty of Chemistry, Regional Campus of International Excellence “Campus Mare Nostrum”, University of Murcia, E-30100 Murcia, Spain; 2 Department of Animal Production, UIC ENZOEM, International Agrifood Campus of Excellence (ceiA3), University of Cordoba, Campus de Rabanales, 14071 Córdoba, Spain; 3 Sustainability and Quality Group of Fruit and Vegetable Products, Murcia Institute of Agricultural and Environmental Research and Development, C/Mayor s/n. La Alberca, 30150 Murcia, Spain

**Keywords:** Dispersive magnetic solid-phase extraction, HPLC-MS, Mycotoxins, Magnetic polypyrrole microcomposite, Natural grass analysis

## Abstract

An analytical methodology based on the combination of dispersive magnetic solid-phase extraction (DMSPE) and liquid chromatography-mass spectrometry (LC-MS) is proposed to explore the occurrence of 13 mycotoxins (aflatoxins B_1_, G_1_, B_2_, and G_2_; deoxynivalenol; T-2 toxin; ochratoxin A; HT-2 toxin; enniatins A, A_1_, B, and B_2_; and beauvericin) and their derivatives in natural grass samples. Magnetic microparticles (Fe_3_O_4_) coated with polypyrrole (PPy) polymer were used in DMSPE sample treatment as adsorbent phase, and Fourier-transform infrared spectroscopy, field emission scanning electron microscopy, and energy dispersive X-ray spectroscopy have been used for its characterization. The experimental parameters influencing the adsorption and desorption steps of DMSPE have been optimized. Method validation has been carried out obtaining limits of quantification between 0.07 and 92 μg kg^−1^ corresponding to enniatin B or A_1_ and DON, respectively. A total of 83 natural grass samples from 8 *dehesa* farms were analysed. Enniatin B was found in all the samples (0.29 to 488 μg kg^−1^ concentration range) followed by enniatin B_1_ (92.8% of the samples) with a 0.12–137 μg kg^−1^ concentration range. Moreover, co-occurrence of mycotoxins was studied and between 2 and 5 mycotoxins appeared simultaneously in 97.6% of the samples. Distribution of the contamination according to natural grass location was also investigated.

**Graphical abstract:**

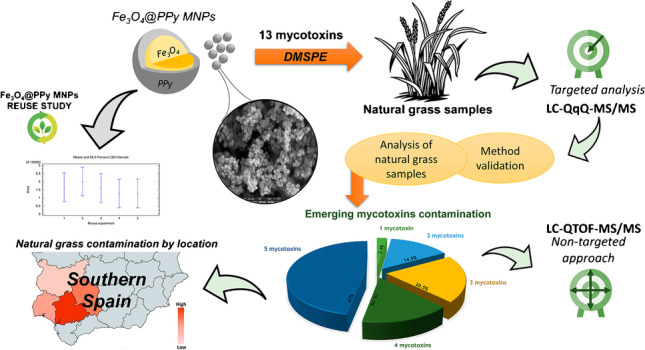

**Supplementary Information:**

The online version contains supplementary material available at 10.1007/s00604-023-05763-6.

## Introduction

The ingestion of mycotoxins in animals can cause renal, hepatotoxic, mutagenic, teratogenic, or carcinogenic effects. Moreover, they can cause suppression of immune system, retardation of growth, lower weight gain, decrease in production of meat, eggs, or milk, and fertility problems including abortions. Some mycotoxins are neurotoxic, producing paralysis and convulsions, and although subacute and chronic effects are more frequent, mycotoxins are also related to high mortality [1]. In addition, the consumption of several mycotoxins together can have a synergistic, additive, antagonistic, or potentiating effect on animal health.

Natural grasses are essential resources for animal feeding being susceptible to contamination by mycotoxins. In general, less information is available regarding mycotoxin levels in natural grass compared to the data in grains and conserved feeds. A recent review has summarized the occurrence of mycotoxins in fresh pastures and conserved forage, concluding that *Fusarium* mycotoxins are the most frequent in this type of matrices. Zearalenone (ZEN) was the most prevalent mycotoxin followed by deoxynivalenol (DON) and T-2 and HT-2 toxins [2]. It should be noted that most of the studies focus on the monitoring of regulated mycotoxins in animal feed (aflatoxin B1 (AFB_1_), DON, ZEN, fumonisins (FBs), ochratoxin A (OTA), and T-2 and HT-2 toxins) [3, 4], and to date, there is scarce information on the contamination of pastures by emerging mycotoxins (enniatins (ENNs) and beauvericin (BEA)) or modified mycotoxins, derivatives of the main mycotoxins whose structure has changed due to their binding to other matrices, or to the modification of their basic structure caused by chemical or biological modifications [5].

Given the potential for mycotoxin contamination of pastures and forages and the frequent co-occurrence of fungal metabolites in these animal feed, multiple mycotoxin detection methods are in demand. At present, liquid chromatography (LC) coupled to high-resolution mass spectrometry (HRMS) or tandem mass spectrometry (MS/MS) sensitive analytical devices has been more widely applied for multiclass mycotoxin determination in forage, silage, and pasture samples [5–19]. To a lesser extent, enzyme-linked immunoassay (ELISA), thin layer chromatography (TLC), electrochemical assay, and gas chromatography (GC) have also been used for the same purpose with high accuracy and precision [20–23].

Concerning the sample treatment for multiclass mycotoxin assessment in pastures and forages, in recent years, mainly QuEChERS (acronym of Quick, Easy, Cheap, Effective, Rugged, and Safe), solid-phase extraction (SPE), and extraction with organic solvents [5–7, 9–12, 15–19, 24] have been used. Dispersive magnetic solid-phase extraction (DMSPE) is a novel miniaturized technique that simplifies and reduces sample preparation stage and time required with respect to conventional SPE. DMSPE provides great results for analyte recovery and preconcentration due to the high contact surface generated by dispersing the sorbent into the sample matrix [25].

In case of mycotoxin contamination of pasture, the choice of a low-cost, rapid, and high-throughput analytical approach is crucial. In this respect, nanotechnology plays a fundamental role in the design and construction of promising materials, and therefore, the use of a suitable magnetic sorbent is key when using the DMSPE technique. So far, different nanomaterials have been used for the determination of specific groups of mycotoxins, including multi-walled carbon nanotubes for AFB_1_ and ZEN extraction from wheat flour and for main aflatoxins (AFs) (aflatoxins B_1_, B_2_, G_1_ and G_2_). In addition, other multi-walled carbon nanotubes based materials have also been used for the determination of aflatoxins M_1_ (AFM_1_) and M_1_ (AFM_2_), OTA, ZEN, zearalanone (ZAN), α-zeralanol, β-zeralanol, α-zeralenol and β-zeralenol in liquid milk [26, 27], as well as core-shell nanomaterials in the form of covalent or metal-organic frameworks for the analysis of maize (AFs, ochratoxins and ENNs) or liquorice (AFG_1_, AFB_1_, sterigmatocystin, ZEN and OTA) [28, 29]. Other magnetic nanoparticles (MNPs) based on ferrite cores with nonporous silica shell have been used for fumonisin B1, ZEN, and OTA preconcentration from vegetable oil [30], or with cellulose shell for ENNs and BEA from paprika samples [31].

The main objective of this work is the development of an analytical methodology based on the combination of DMSPE and LC-MS for the determination of 13 mycotoxins derived from *Aspergillus* and *Fusarium* (AFB_1_, AFB_2_, AFG_1_, AFG_2_, OTA, enniatin A (ENNA), enniatin B (ENNB), enniatin A1 (ENNA_1_), enniatin B1 (ENNB_1_), DON, HT-2, BEA, and T-2 toxin) and their derivatives including modified mycotoxins in natural grass samples from different Spanish *dehesa* farms, with the aim of studying its occurrence in this type of little-explored matrices. To the best of our knowledge, this is the first application of a DMSPE-based method not only for the analysis of natural grass samples but also for the quantification of 13 mycotoxins of high interest and belonging to different families, resulting in a multiclass mycotoxin assessment tool not previously reported and of great novelty. The combination of low- and high-resolution MS allows both targeted and non-targeted analysis enabling the quantification of 13 mycotoxins but also the monitoring of other derivatives for which there are no standards, with the aim to obtain a better understanding of the occurrence of mycotoxins in the natural grass samples.

## Materials and methods

### Reagents and standards

Individual mycotoxin standards of AFG_1_, AFB_1_, AFG_2_, AFB_2_, OTA, ENNB, ENNA, ENNB_1_, ENNA_1_, BEA, and DON were acquired from Sigma-Aldrich (St. Louis, MO, USA). HT-2 and T-2 were provided by n’TOX (Saint Jean d'Illac, France). All mycotoxins were prepared as separated stock solutions at 1000 μg mL^−1^ in acetonitrile (MeCN) and placed in storage at −20 °C. Ethyl acetate (EA), methanol (MeOH), ethanol (EtOH), and MeCN of chromatographic grade were supplied by ChemLab (Zedelgem, Belgium).

For the synthesis of the microcomposite, pyrrole, iron (III) chloride hexahydrate (FeCl_3_·6H_2_O), ammonia solution, iron (II) chloride tetrahydrate (FeCl_2_·4H_2_O), sodium perchlorate, and sodium hydroxide reagents were all acquired from Sigma-Aldrich. Milli-Q water was generated by a Millipore Milli-Q system (Bedford, MA, USA).

Formic acid and ammonium acetate were used for the mobile phase. In addition, during the DMSPE procedure optimization, sodium chloride was used. All the reagent above mentioned were supplied by Sigma-Aldrich.

Before chromatographic analysis, sample extract filtration was carried out using 0.22 μm × 25 mm nylon syringe filters purchased from Agilent Technologies (New York, USA).

### Instrumentation and software

The targeted analysis was carried out using a 1200 series high-performance LC from Agilent Technologies coupled to an Agilent G6410A triple quadrupole (QqQ) mass spectrometer furnished with an ionization source based on electrospray (ESI). Chromatographic separation of mycotoxins was performed using an InfinityLab Poroshell 120 EC-C18 column (4.6 mm of inner diameter, 2.7 μm of particle size, and 150 mm of length). Multiple reaction monitoring (MRM) mode was used for MS/MS detection with ESI in positive mode.

For non-targeted analysis, an Agilent 1290 Infinity II Series HPLC (Agilent Technologies, Santa Clara, CA, USA) with a high-speed binary pump (thereby comprising the UHPLC system) was used. Separations were carried out using a ZORBAX RRHD Eclipse Plus C18 column (2.1-mm inner diameter, 1.8-μm particle size, and 100-mm length) and a 0.3-μm Agilent Technologies inline filter. Detection was performed with an Agilent 6550 Quadrupole Time of Flight (QTOF) mass spectrometer provided with an Agilent jet stream dual electrospray (AJS-Dual ESI) source.

For sample processing, an IKA A11 basic analytical mill (Wilmington, USA), an orbital shaker IKA-KS-130-Basic (Staufen, Germany), and an air-drying system (XcelVap) from Horizon Technology (Salem, USA) were used. A magnet block consisted of Nd-Fe-B with a 33-kg strength, 50 × 15 × 15 mm dimensions, and weight of 86 g was employed. Such magnets were acquired in Supermagnete (Gottmadingen, Germany).

An ApreoS Thermo field emission scanning electron microscopy (FESEM) system from ThermoFisher Scientific (Massachusetts, USA) and an EDAX Ametek (Mahwah, USA) were used for image data acquisition and energy dispersive X-ray spectroscopy (EDS) analyses. A Jasco FT/IR-4600 spectrophotometer obtained from Jasco Corporation (Japan) was used for Fourier-transform infrared spectroscopy (FTIR). Data acquisition was performed with Jasco Spectra Manager software, and spectra were saved as JCAMP-DX files. Malvern Zetasizer Nano ZS (Malvern Instruments Ltd., UK) instrument was used for dynamic light scattering (DLS) measurements. XPowder X software (Granada, Spain) was used for X-ray diffraction pattern analysis using PDF2.DAT database of the International Centre for Diffraction Data (ICDD).

Agilent MassHunter Quantitative Analysis and Profinder were the software used for mycotoxin and metabolite identification and quantification. SigmaPlot 13.1 (Systat Software Inc., San Jose, CA, USA) was used for data treatment. Statgraphics Centurion XV version 15.1.02 was used for multivariate experimental designs.

### Samples

Different natural grass samples from 8 different *dehesa* farms (pilot farms within the LIFE project LiveAdapt) located in 4 Spanish provinces with Mediterranean climate (Sevilla, Huelva, Córdoba, and Badajoz) were obtained, specifically, a total of 83 samples (Supplementary Table S1). The most frequent natural pastures of the *dehesa* are annual grasses on shallow and poor acid soils. These pastures are composed of short species of the communities *Helianthemetalia*, *Thero-Brometalia*, and *Sisymbrietalia*, with premature drying at the end of spring [32].

The samples came from grazing exclusion cages of 1 m^2^ and were collected by mid-May. All samples were mixtures of freshly mowed natural grasses dehydrated at 60 °C for 48 h.

Samples were ground and transferred to sterile plastic containers and stored at room temperature until analysis. The average dry matter (DM) content of the natural grass samples was also calculated as follows: DM (%) = 100 * [(wet sample weight − dry sample weight)/wet sample weight].

### Fe_3_O_4_@PPy microcomposite synthesis

The synthesis of Fe_3_O_4_@PPy microcomposite was performed as reported by Asgharinezhad et al. [33] with slight modifications and is presented in the Supplementary Material. A summary scheme of the synthesis can be seen in Fig. S1.

### Sample preparation and extraction

An amount of 0.5 g of the homogenized ground sample was weighed into a 15-mL polypropylene tube and 10 mL of ultrapure water containing 2% m/v NaCl and 400 μL Fe_3_O_4_@PPy microcomposite suspension was added, and the resulting mixture was subjected to orbital shaking at room temperature for 15 min. Afterwards, external magnetically attraction with neodymium magnet was performed and the supernatant was discarded. To desorb the mycotoxins, 2 mL of EA was added to the enriched magnetic material, and orbital shaking was performed for 10 min at ambient temperature. Separation of the microcomposite from the supernatant was then again performed using the magnet. Finally, the collected EA supernatant solution was evaporated under a N_2_ stream (1200 mbar) until dryness at 35 °C, reconstituted in 500 μL of (50:50, v/v) MeOH/H_2_O mixture and submitted to vortex agitation for 1 min. Then, filtration with 0.2-μm nylon filter of the reconstituted extract was carried out prior injection.

### LC-QqQ-MS/MS analysis

The 13 parent mycotoxins were eluted using a mobile phase A consisting of 0.1% *v*/*v* HCOOH and 2-mM HCOONH_4_ aqueous solution and a mobile phase B consisting of 0.1% *v*/*v* HCOOH in MeOH. The elution gradient was set as follows: 0–20 min, 30% B–99% B; 20–35 min, 99% B; 35–37 min, 99% B–30% B; and 37–45 min, 30% B. The mobile phase flow-rate was set at 0.5 mL min^−1^. Sample injection volume was 20 μL. Gas temperature, capillary voltage, nebulizer pressure, and gas flow were 350 °C, 3000 V, 40 psi, and 9 L min^−1^, respectively. Collision energies (CE) between 4 and 100 V and fragmentor voltages from 120 to 180 V were evaluated. Table S2 shows the MS conditions used for the mycotoxin determination.

### LC-Q-TOF-MS/MS analysis

Mobile phases consisted of (95:5, *v*/*v*) water/MeOH (solvent A) and (95:5, *v/v*) MeOH/water (solvent B), both containing 0.3% *v*/*v* HCOOH as ionization agent and 5-mM HCOONH_4_ and applying a flow rate of 0.4 mL min^−1^. The elution gradient was the following: linear gradient from 0 to 99% solvent B for 20 min; then, solvent B ratio decreased in 1 min to 0%; and finally, an isocratic step of 5-min duration maintaining a 0% ratio of solvent B. ESI source parameters operated in positive mode were the following: nebulizer gas pressure of 30 psi, drying gas temperature and flow rate of 130 °C and 16 L min^−1^, respectively, and sheath gas flow rate and temperature of 11 L min^−1^ and 300 °C, respectively. Capillary voltage was 4000 V, while the nozzle, fragmentor, and octopole voltages were set at 500, 360, and 750 V, respectively. Data-dependent analysis approach for MS detection was performed by using auto MS/MS mode in a 100–1200 *m/z* range.

## Results and discussion

### Microcomposite characterization

To characterize the synthesized Fe_3_O_4_@PPy composite in terms of nature, surface morphology, and elemental composition, field emission scanning electron microscopy (FESEM) and EDS techniques were used.

**Fig. 1:**
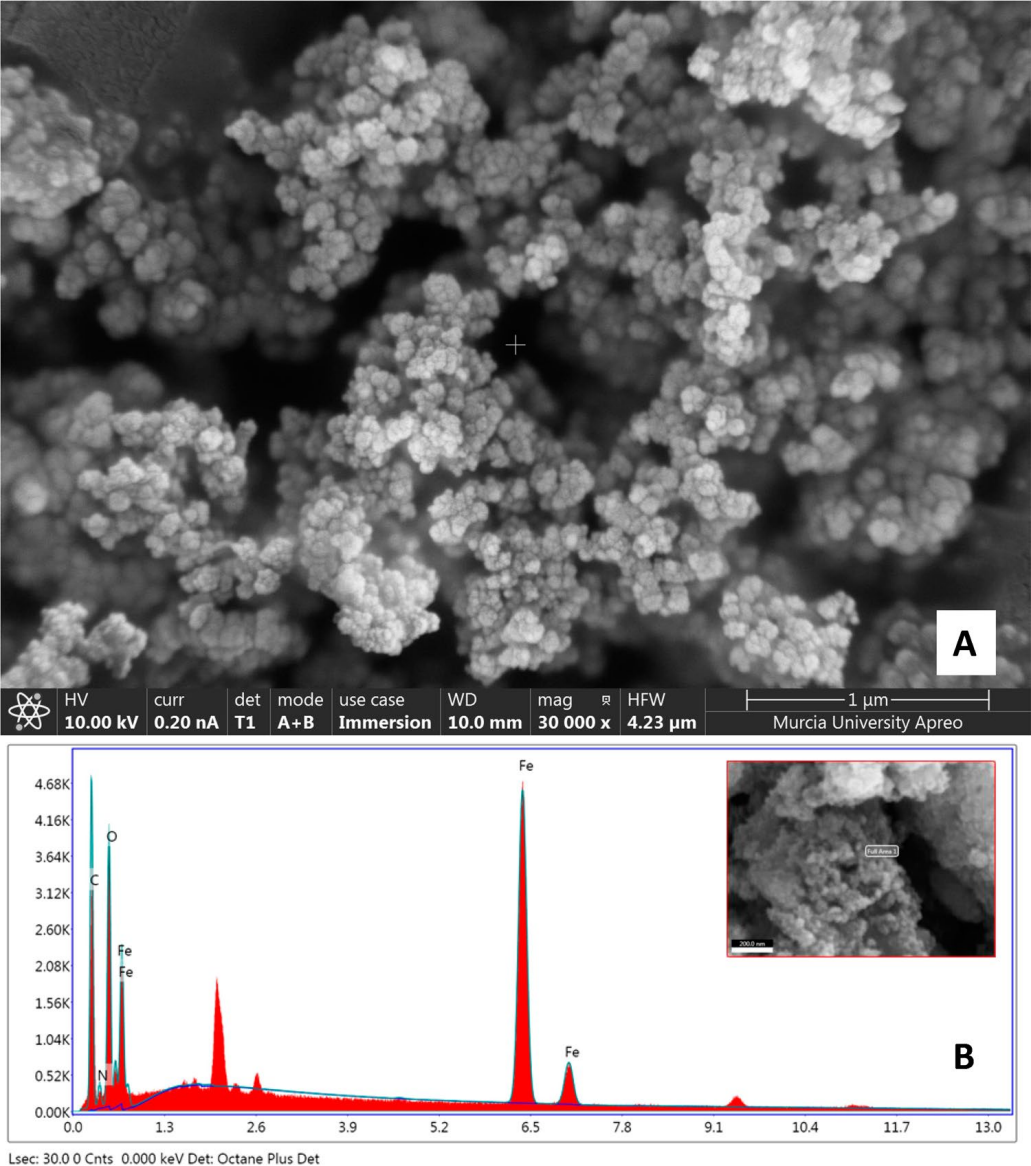
Field emission scanning electron microscopy (FESEM) image (**A**) and energy dispersive X-ray spectroscopy (EDS) spectrum (**B**) of Fe_3_O_4_@PPy microcomposite

The composite was assembled on special SEM aluminium holders, heated at 60 °C for 10 min till dehydration and coated in a vacuum sputter with 5 nm of platinum. FESEM acquired image is presented in Fig. 1A and provides the morphology of Fe_3_O_4_@PPy material at 1-μm resolution. Noticeably, the microcomposite consists of many spherical grains with a high homogeneity grade regarding particle size and distribution.

For EDS analysis, an accelerating voltage of 20 kV was employed. Peaks related to C, Fe, O, and N atoms are shown in the EDS spectrum (Fig. 1B). The weight and atomic percentages were calculated at 3 different points of the material. Quantitative analysis average values obtained were 41.56 and 59.28% for C, 30.66 and 9.76% for Fe, 22.42 and 24.29% for O, and 5.37 and 6.66% for N, which corresponded to weight and atomic percentages, respectively. In the right corner of Fig. 1B, it can be seen a FESEM image which corresponds to the Fe_3_O_4_@PPy micromaterial area where the measurements were carried out.

For FTIR spectroscopic measures, the spectrophotometer used was equipped with an ATR PRO ONE attenuated total reflection accessory that uses a monolithic diamond crystal to provide high optical processing performance. It features a “torque-limited” pressure applicator to press the sample into good contact with the diamond. Data acquisition was performed over a range of 4000 to 250 cm^−1^ with a resolution of 4 cm^−1^. Thirty-two accumulations were recorded for each sample with a total measurement time of 38 s. Fig. S2 shows the FTIR spectra of Fe_3_O_4_@PPy microcomposite, PPy, and Fe_3_O_4_. The curve (a) of the Fe_3_O_4_@PPy microcomposite seemingly reveals peaks of both the PPy and Fe_3_O_4_ components shown in curves (b) and (c), respectively. The peak observed at 541 cm^−1^ in Fe_3_O_4_ and Fe_3_O_4_@PPy spectra corresponded to the absorption peak of the Fe-O group [34]. The peaks at 924 cm^−1^ and 1205 cm^−1^ are observed at almost the same place in both PPy and Fe_3_O_4_@PPy curves and are attributed to C–C out-of-plane ring deformation and C–N stretching vibrations, respectively [35]. Furthermore, the peak observed at 1044 cm^−1^ in PPy and Fe_3_O_4_@PPy spectra shows the C-O-C absorption function of a shift in the stretching vibrations when PPy was added, which is due to energy changes and the PPy-Fe_3_O_4_ interaction [34, S36]. Besides that, the appearance of a peak at 3393 cm^−1^ is attributed to the existence of –OH groups on the surface of the Fe_3_O_4_ [S37], which is a vibration of stretching and bending [34].

In addition, DLS and X-ray diffraction (XRD) techniques were used to carry out a more thorough characterization of the material. DLS measurements were performed by adding 5, 15, and 25 mg of solid Fe_3_O_4_@PPy to 2 mL of water. A single cycle of 13 runs was applied and no equilibration time was required. Under these conditions, the hydrodynamic diameter (d_h_) values obtained were 509.9, 512.6, and 523.5 nm for each amount of micromaterial measured, which means an average diameter of 515.3 nm (Fig. S3). XRD data revealed the presence of two types of magnetic iron oxide: magnetite (Fe_3_O_4_) and maghemite (γ-Fe_2_O_3_) with weight percentages around 61% and 39%, respectively. The standard X-ray diffraction peaks (2ϴ = 30.18°, 35.56°, 43.14°, 57.20°, and 62.79°) which can be assigned to maghemite or magnetite match with those observed in the spectrum depicted in Fig. S4.

### DMSPE procedure optimization

For the optimization of the DMSPE procedure, the factors affecting the stages of adsorption and desorption were investigated in detail.

To carry out the preliminary experiments, a pool of different mycotoxin-free natural grass samples, which were previously analysed, was made. The pool was done by adding 5 g of each of the samples, and the resulting mixture was left to stand for 24 h at room temperature. Thus, initial experiments were conducted using 0.5-g pooled natural grass sample spiked at 100 μg kg^−1^ with the five emerging mycotoxins (ENNA, ENNB, ENNA1, ENNB1, and BEA), the four AFs (AFB1, AFG1, AFB2, and AFG2), and OTA and at 500 μg kg^−1^ with HT-2, DON, and T-2 toxin.

The influence of the nature of the magnetic material on MSPE efficiency was studied by adding 30 mg of each material type assayed to 0.5 g of natural grass suspended in 10 mL of water, which was followed by an adsorption time of 30 min and the addition of 1.5 mL of MeCN and orbital shaking during 8 min for mycotoxin desorption. The appropriate choice of sorbent is essential for the isolation of analytes and depends on the nature of the particles and of the sample being tested. Seven different magnetic materials were evaluated using ferrite (Fe_3_O_4_) core with different coating materials: polypyrrole (PPy), cellulose, silver (Ag), oleic acid, multi-walled carbon nanotubes (MWCNTs), 3-aminopropyl-triethoxysilane (APTS), and MWCNTs/PPy. Coating with PPy and APTS was tested due to their environmental and mechanical steadiness, ease of synthesis, regeneration, and low cost [S38]. Fe_3_O_4_@cellulose (S39) was tested since this coating material presents a high potential for biodegradation. Fe_3_O_4_@Ag (S40) was examined because the presence of heteroatoms in the structure of mycotoxins makes them capable of interacting with silver, and this potential interaction between amino groups of other analytes with Ag nanoparticles has been previously investigated (S41). Moreover, the strongest benefit of oleic acid coating lays on the chemical bond between the iron oxide amorphous nanoparticles and the carboxylic acid group (S42). Finally, Fe_3_O_4_@MWCNTs [33] was considered because of its high efficiency, porosity, and large surface area.

Fig. S5 shows that signals increased for the 13 mycotoxins when the Fe_3_O_4_@PPy microcomposite was used, the other tested materials providing lower enrichment. This is probably due to the intense π-π stacking, hydrogen bonding, and electrostatic adsorption interactions between the PPy polymer and the mycotoxins. Then, PPy coating was compared in Fe_3_O_4_ and cobalt ferrite (CoFe_2_O_4_) core as the corrosion resistance, excellent stability and high coercive force, and saturation magnetization of CoFe_2_O_4_ core have been previously described (S43, S44). However, best results were obtained when the core was made of Fe_3_O_4_.

Once the nature of the extractant phase was optimized, other parameters influencing the DMSPE adsorption step were studied, such as the effect of directly adding the solid material or suspended in water, the amount of material, the adsorption time, and the ionic strength of the sample solution.

Whether the addition of the Fe_3_O_4_@PPy microcomposite as an aqueous suspension or as solid material influences the sensitivity of the method was examined. This comparison was carried out by adding 102 μL of an aqueous Fe_3_O_4_@PPy suspension (976 mg mL^−1^ MNP concentration) or 100 mg of Fe_3_O_4_@PPy solid material. Preconcentration was enhanced when the microcomposite was added in suspension form as can be seen in Fig. S6. It may be a result of a weaker MNP assembly in suspension in comparison to their directly addition to the sample solution. For this reason, the suspension form of the adsorbent phase was used.

The other variables involved in the adsorption step, MNP suspension volume, adsorption time, and ionic strength were optimized together because their effect is closely related to each other. Thus, using the peak area as analytical response, a face-centred surface response multivariate method design (2^3^ + star) with three spaced central points was performed. A total of 17 runs were carried out to create the response surface by evaluating the following ranges for each factor: Fe_3_O_4_@PPy suspension volume (100–400 μL), adsorption time (15–45 min), and NaCl concentration (0–10% m/v).

As expected, the micromaterial suspension volume significantly affected the analytical signal for all compounds, whereas the adsorption time and NaCl percentage only affected OTA and ENNA_1_, respectively. Determination coefficients (*R*^2^) resulted in a range of 87.9–91.1%, proving the suitability of the design. Including the analytical signal of all compounds, a robust joint desirability study was performed, being the optimal conditions for the variables involved in the adsorption step: 400 μL of Fe_3_O_4_@PPy microcomposite suspension, 15 min of adsorption time, and 2% m/v NaCl (Fig. S7).

Four solvents with different polarities, being EA, chloroform, MeCN, and MeOH, were further investigated for the desorption of mycotoxins from the PPy-magnetic material. The results showed that EA gave the highest desorption efficiency, followed by MeCN, MeOH, and chloroform. Consequently, EA was selected as the desorption solvent.

To evaluate the influence of desorption time and EA volume, a face-centred multivariate method design was again used to assess the potential effect of these variables and interaction between them. For this purpose, 10 mL of water containing 2% m/v NaCl were added to 0.5 g of pooled natural grass sample, fortified at 100 μg kg^−1^ with ENNs, AFs, BEA, and OTA and at 500 μg kg^−1^ with HT-2, DON, and T-2. Then, 400 μL of MNP suspension was added, and the mixture was submitted to orbital shaking for 15 min to carry out analyte adsorption on the MNP surface. In this case, the response surface was created carrying out a total of 11 runs by evaluating each factor at three levels. Desorption time was studied between 1 and 15 min and EA volume between 1 and 3 mL. The mycotoxins that showed significant differences were AFB_1_, AFG_1_, AFB_2_, AFG_2_, ENNA, and OTA, whose *R*^2^ coefficients were in a range of 87.4–96.3%. Including only those mycotoxins with significant differences, the optimal conditions for desorption step were 2 mL of EA and 10 min of orbital shaking (Fig. S8).

### Validation of the analytical method

For method validation, a sample of natural grass previously checked to be free from the studied mycotoxins was used. Before applying the analytical procedure, this sample was fortified at different concentration levels with the mycotoxins, homogenized and left to stand for 1 h in the dark at room temperature to allow interaction between mycotoxins and natural grass matrix.

Matrix-matched calibration graphs were set by fortifying natural grass samples at seven concentration levels which were injected in duplicate. AFs, OTA, BEA, and ENN calibration concentration levels varied from 0.07 to 100 μg kg^−1^, and concentrations from 17 to 750 μg kg^−1^ were carried out for DON, HT-2, and T-2, depending on the mycotoxin. Resolution achieved can be seen in Fig. S9. Table 1 shows the calibration parameters obtained after applying least-square regression. Linearity in the studied ranges was demonstrated as regression coefficient (*R*^2^) values were greater than 0.985 in all cases.

**Table 1:** Method validation data for mycotoxin determination in natural grass samples

Analyte	Linear range (μg kg^−1^)	Linearity, *R*^2^	LOD (μg kg^−1^)	LOQ (μg kg^−1^)
DON	92–750	0.990	27	92
AFG_2_	1.7–100	0.989	0.51	1.7
AFG_1_	0.95–100	0.998	0.28	0.95
AFB_2_	1.3–100	0.996	0.38	1.3
AFB_1_	0.61–100	0.998	0.18	0.61
HT-2	37–750	0.994	11	37
T-2	17–750	0.995	5.3	17
OTA	1.9–100	0.985	0.57	1.9
ENNB	0.07–100	0.993	0.02	0.07
BEA	0.09–100	0.994	0.03	0.09
ENNB_1_	0.09–100	0.997	0.03	0.09
ENNA_1_	0.07–100	0.987	0.02	0.07
ENNA	0.08–100	0.996	0.02	0.08
	Trueness, % RSD (*n* = 9)		SSE (%)	
	Low level	High level		
DON	90 (4.2)	98 (5.9)	42.0	
AFG_2_	96 (2.1)	97 (5.3)	52.4	
AFG_1_	110 (5.9)	100 (2.8)	57.7	
AFB_2_	90 (4.4)	105 (5.8)	56.4	
AFB_1_	108 (6.5)	110 (2.1)	59.9	
HT-2	82 (7.1)	106 (1.9)	66.3	
T-2	76 (2.8)	92 (8.7)	47.1	
OTA	78 (2.7)	103 (8.1)	42.4	
ENNB	107 (4.5)	106 (1.8)	91.9	
BEA	95 (5.3)	97 (1.9)	89.6	
ENNB_1_	102 (4.7)	104 (6.1)	92.2	
ENNA_1_	99 (3.1)	104 (2.6)	89.5	
ENNA	104 (5.0)	108 (3.2)	88.0	
	Repeatability, % RSD (*n* = 6)		Intermediate precision, % RSD (*n* = 18)	
	Low level	High level	Low level	High level
DON	5.4	3.7	7.3	6
AFG_2_	1.5	2.6	5.4	6
AFG_1_	3.8	4.1	8.1	5.9
AFB_2_	4.8	8.5	7.3	9.5
AFB_1_	5.6	5.5	7.8	8.8
HT-2	2.9	6.8	8.7	10.2
T-2	3.2	5.1	4.2	10.2
OTA	4.7	6.1	6.4	7.1
ENNB	2.8	5.8	3.6	7.3
BEA	4.9	5.4	7.1	7.8
ENNB_1_	2.4	3.6	4.5	5.6
ENNA_1_	2.6	4.8	4.9	8.4
ENNA	3.5	3.6	6.8	6.9

*LOD*, limit of detection (S/N = 3); *LOQ*, limit of quantification (S/N = 10); *SSE*, magnitude of signal suppression/enhancement

Low level: 5 μg kg^−1^ for AFs, OTA, BEA, and ENNs; 300 μg kg^−1^ for DON, HT-2, and T-2

High level: 25 μg kg^−1^ for AFs, OTA, BEA, and ENNs; 500 μg kg^−1^ for DON, HT-2, and T-2

Adequate values were obtained for all mycotoxins. LODs varied between 0.02 μg kg^−1^ for ENNB, ENNA, or ENNA_1_ and 0.57 μg kg^−1^ for OTA, not considering DON, for which a significantly higher LOD was obtained (27 μg kg^−1^). By comparison, LOQs were in the 0.07–92 μg kg^−1^ range, corresponding to ENNB or ENNA_1_ and DON, respectively (Table 1). The limits obtained allow the correct determination of the mycotoxins studied, complying with the limits established in the legislation [3, 4].

The magnitude of signal suppression/enhancement (SSE) allowed to evaluate the presence of matrix interferences. For this purpose, slopes obtained by linear calibration built in a blank matrix and on pure solvent were compared. Consequently, SSE effect was quantified as follows: SSE (%) = 100 * (slope for spiked cleaned-up extract/slope for spiked matrix-free solvent). SSE values in the 42.0–92.2% range were obtained (Table 1). These results showed SEE values below 100% in all cases, revealing signal suppression in the presence of matrix. Specifically, the highest signal suppression occurred for DON, T-2 toxin, and OTA, while the mycotoxins whose SEE values were closest to 100% were ENNs and BEA. Given this, the high matrix effect for some of the studied mycotoxins justified the need to perform matrix-matched calibrations for quantification purposes.

Trueness of the proposed method was evaluated by conducting recovery studies. Hence, fortification at two concentration levels was carried out: 5 μg kg^−1^ for the four AFs, OTA, BEA, and the four ENNs and 300 μg kg^−1^ for DON, HT-2, and T-2 and the second level stated at 25 μg kg^−1^ for AFs, OTA, BEA, and ENNs and at 500 μg kg^−1^ for DON, HT-2, and T-2. Accordingly, the apparent recovery was calculated as follows: 100 * [concentration determined/actual (spiked) concentration]. The mean recoveries of nine experiments, each concentration level was prepared and injected in triplicate, are shown in Table 1. Recoveries were in the 76–110% range and the relative standard deviation (RSD) values ranged from 1.8 to 8.7%.

Precision was assessed in terms of repeatability and intermediate precision. The whole procedure was applied to perform experiments which consist of sample spiked at two concentration levels in triplicate along the same day to evaluate repeatability. Low and high concentration levels were set at 5 and 25 μg kg^−1^ for AFs, OTA, BEA, and ENNs, as well as 300 and 500 μg kg^−1^ for DON, HT-2, and T-2. RSD values in a range of 3.6–8.7% were obtained (Table 1).

In addition, analysis on three different days of three samples fortified at the same two concentration levels was used to establish intermediate precision. In all cases, RSD was calculated, and the results were below 10.2%, demonstrating the good precision of the method.

### Occurrence of mycotoxins in natural grass samples

Mycotoxin occurrence in natural grass samples has been assessed by performing the analysis of 83 samples from 8 *dehesa* farms using the developed method. Duplicates of the samples were prepared, processed by the described DMSPE method for mycotoxin extraction, and injected into the chromatographic system. The samples were treated in duplicate and injected into the LC-QqQ-MS/MS system.

Among the 13 mycotoxins studied, all the samples resulted positive (considering samples with concentrations above the LOQ) for emerging mycotoxins with the following incidence: ENNB (100%), ENNB_1_ (92.8%), ENNA (51.8%), ENNA_1_ (71.1%), and BEA (74.7%). However, none of the samples tested positive for the other examined mycotoxins (DON, AFB_1_, AFB_2_, AFG_1_, AFG_2_, HT-2, and T-2). The lack of aflatoxins in natural grasses has been already reported (S45).

A summary of all results obtained for emerging mycotoxin occurrence is shown in Table 2, referring to both dry matter and fresh natural grass. Thus, Table 2 includes number and percentage of positive samples, mean concentration value of positive samples, and 1^st^ and 3^rd^ quartile of positive samples. The mycotoxin quantified at the highest level was ENNB at 488 μg kg^−1^, with an average concentration in the positive samples of 51.1 μg kg^−1^, being these concentrations equivalent to 1154 and 83.7 μg kg^−1^ in terms of wet matter, respectively. Moreover, ENNB was also the most occurring mycotoxin (100%) followed by ENNB_1_ (92.8%), ranging from 0.29 to 488 μg kg^−1^ and from 0.12 to 137.1 μg kg^−1^, respectively, related to dry matter. In the case of BEA and ENNA concentrations, these ranged from 0.10 to 36.5 μg kg^−1^ and 0.07 to 18.6 μg kg^−1^ in terms of dry matter, respectively. On the other hand, the emerging mycotoxin that appeared with the lowest incidence in the samples was ENNA_1_ (51.8%) with a mean concentration of 1.74 μg kg^−1^ and a range of 0.09 to 22.6 μg kg^−1^. Fig. S10 shows the box plot where the statistical data are summarized. As can be seen in Fig. S10, ENNB shows the widest interquartile range, followed by ENNB_1_, while ENNA, ENNA_1_, and BEA are similar, with the lowest point of the 5 emerging mycotoxins being below 0.3 μg kg^−1^ of dry matter. In the case of ENNB, positive asymmetry is also observed, as the part of the box above the median is longer, indicating that the data are concentrated in the lower part of the distribution. In addition, in all cases, some values out of range were found beyond the lower or upper limits.

**Table 2:** Summary of emerging mycotoxins occurrence

	ENNB	ENNB_1_	ENNA	ENNA_1_	BEA
No. of positive samples	83	77	59	43	62
Incidence (%)	100.00	92.8	71.1	51.8	74.7
Referred to dry matter (wet matter)					
Mean (μg kg^−1^) ± SD	51.1 ± 95.4 (83.7 ± 176.6)	7.82 ± 19.5 (11.6 ± 26.4)	2.16 ± 4.21 (3.02 ± 5.47)	1.74 ± 4.79 (2.69 ± 8.50)	2.76 ± 6.57 (4.01 ± 9.34)
1^st^ quartile (μg kg^−1^)	2.26 (3.73)	0.33 (0.53)	0.20 (0.33)	0.12 (0.18)	0.25 (0.35)
3^rd^ quartile (μg kg^−1^)	54.9 (74.6)	6.02 (10.80)	1.40 (2.62)	1.31 (1.83)	2.41 (3.04)

*SD*, standard deviation

**Fig. 2:**
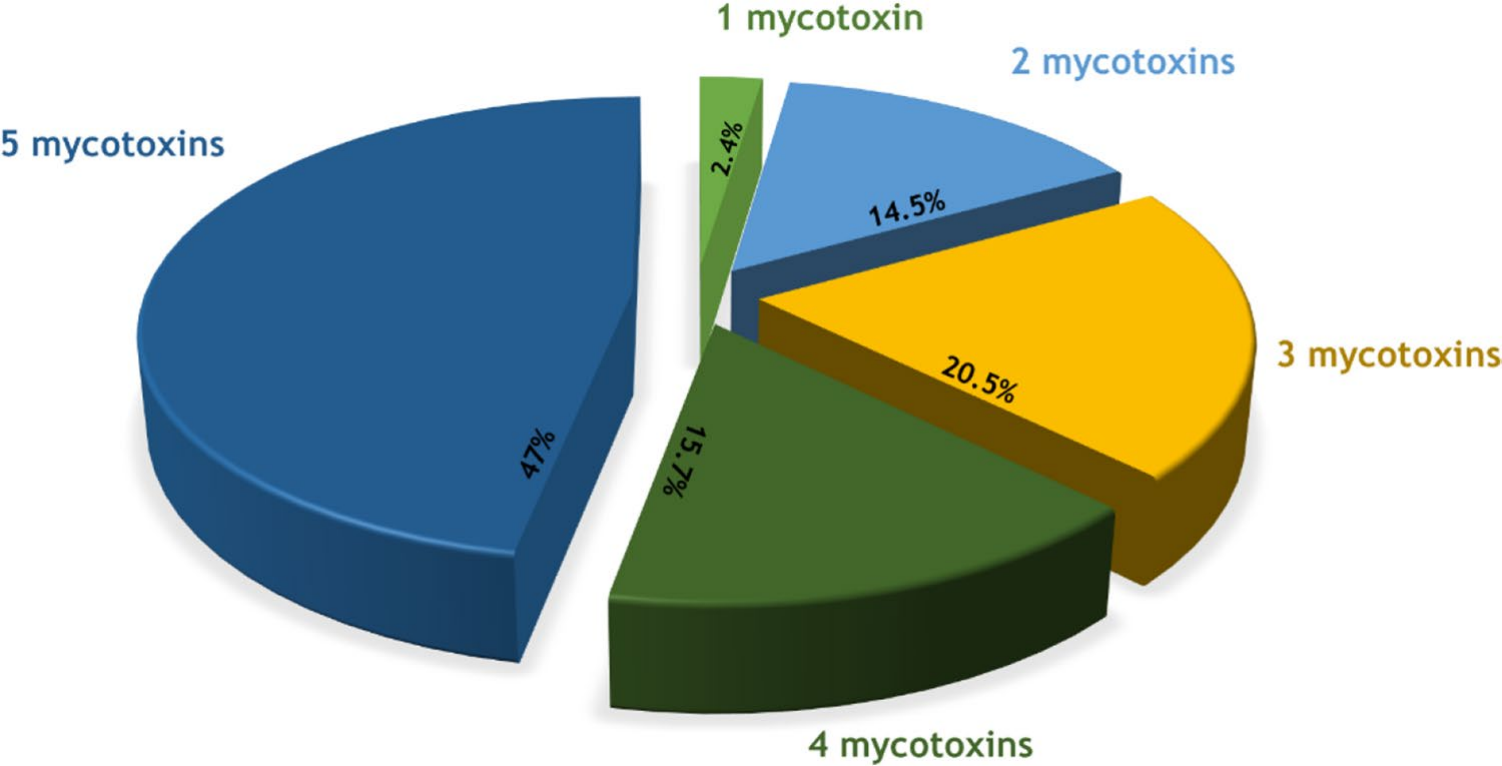
Frequency and co-occurrence of different emergent mycotoxins (ENNA, ENNA_1_, ENNB, ENNB_1_, and BEA) in natural grass samples

In addition, the co-occurrence of the toxins in the samples was also investigated. Thus, 97.6% of the samples contained between 2 and 5 mycotoxins (Fig. 2) and the remaining 2.4% corresponded in all cases to the presence of ENNB singly. This co-occurrence of emerging mycotoxins is observed in the form of different combinations. The most frequent combinations are, in the case of 2 mycotoxins, ENNB+ENNB_1_ (9.6%); when 3 co-occur, ENNB+ ENNB_1_+BEA (10.8%), and when the combination is quaternary in 12.1%, the most frequent is ENNB+ENNB_1_+ENNA_1_+BEA. On the other hand, the 5 emerging mycotoxins co-occur in most of the positive samples, accounting for 47% of the total number of positive natural grass samples.

The presence of emerging mycotoxins and their co-occurrence have been previously reported in animal feed [15], and similar results have been obtained, with ENNB being again the emerging mycotoxin with the highest incidence and the co-occurrence of ENNB+BEA+ENNB_1_ being the most frequent ternary combination.

Then, the contamination obtained by emerging mycotoxins was evaluated according to the province of origin of the natural grass. The samples were classified into 4 groups corresponding to the provinces of origin (Sevilla, Huelva, Córdoba, and Badajoz), and one-way analysis of variance (ANOVA) test was performed to evaluate the differences. Significant differences were obtained for ENNA concentration (*p*-value = 0.0282) for natural grass samples from Sevilla province and for BEA (*p*-value = 0.0206) in the natural grass from Badajoz province with respect to the contents found in the rest of the provinces. On the other hand, ENNB, ENNA_1_, and ENNB_1_ showed no significant differences with respect to their location. Finally, Fig. S11 shows a Southern Spain heatmap with the emerging mycotoxin average contamination obtained in the different provinces.

### Non-targeted approach

With the objective of presenting a complete understanding of the occurrence of this class of mycotoxins in natural grass, a non-targeted approach was carried out to determine the occurrence of derivatives of the 13 mycotoxins studied, including modified mycotoxins, for which no reference standards were available.

Samples were prepared by duplicating and analysed using the LC-Q-TOF method. Data processing consisted of peak alignment and deconvolution on the raw data using Agilent Profinder software. Beside 13 parent mycotoxins, a total of 134 derived metabolites were sought (Table S3). Adducts with the ions Na^+^, K^+^, H^+^, and NH_4_^+^ were researched and 384 possible features were obtained. Subsequently, MassHunter Qualitative software was used for selective extraction of MS/MS information of the monitored molecular features. Tentative identification of mycotoxin metabolites was accomplished by comparing the experimental MS/MS fragmentation spectrum with the data stored in the databases (MassBank MS/MS (http://www.massbank.jp), MassBank of North America (https://mona.fiehnlab.ucdavis.edu/), and METLIN MS and MS/MS (https://metlin.scripps.edu)) and in the literature. The identification of emerging mycotoxins ENNA1, ENNA, ENNB1, ENNB, and BEA could be confirmed, no detecting metabolite derivatives in the analysed samples.

### Micromaterial reuse study

A drawback of DMSPE is the requirement for a synthesis step of the magnetic material. This step can delay the analytical procedure as it requires to be performed thoroughly to obtain reproducible results. The possibility of reusing the nanomaterial would be one way to avoid this disadvantage. Consequently, a study of the reusability of the synthesized Fe_3_O_4_@PPy microcomposite for the determination of multiclass mycotoxins in natural grass samples was carried out.

For this study, 400 μL of the Fe_3_O_4_@PPy microcomposite suspension was used to analyse a spiked natural grass at 100 μg kg^−1^ with the five emerging mycotoxins, the four AFs, and OTA and at 500 μg kg^−1^ with DON, HT-2, and T-2 toxin. Optimal adsorption and desorption conditions were applied, and, after this last step, the MNPs were consecutively reused with four other natural grass samples fortified at the same concentration levels.

The results were evaluated jointly, and for this purpose, the sum of the different mycotoxin areas for each experiment was used to perform ANOVA and least significant difference (LSD) tests to compare the difference reuse experiments. The results are shown in Fig. S12. Even though a slight MNP mass loss during sample treatment was assumed, the results confirmed that the material can be reused up to five times. However, it was decided not to continue reusing the MNPs from the fifth experiment as it was observed that the loss of microcomposite was already significative, and it could not be collected completely.

### Comparison with previously reported methods

The analytical characteristics of the developed method based on the use of Fe_3_O_4_@PPy microcomposite combined with LC-MS/MS were compared with previously reported nanomaterial-based chromatographic methods.

As shown in Table S4, the Fe_3_O_4_@PPy microcomposite had one of the best performances in the determination of multiclass mycotoxins, allowing the preconcentration of 13 mycotoxins and being one of the two methods that allowed the simultaneous determination of more mycotoxins and the only one that allowed the determination of DON, T-2, and HT-2 toxins, in addition to the main aflatoxins and the emerging mycotoxins.

Furthermore, sample preparation time (25 min) is comparable or better than extraction times previously reported, which are in the 15 min to 1 h and 10 min range [26–30]. In terms of analysis results, it can be observed that the proposed method showed comparable or higher validation parameters to the previously reported methods for other magnetic adsorbents.

However, although other existing methods required less adsorbent consumption in terms of solid material, the proposed method is the first one in which the micromaterial is added as a suspension, thus saving the time needed to dry the nanoparticles, which is very long. A drawback of DMSPE procedure is the requirement for a synthesis step of the magnetic material.

In conclusion, the proposed method has proven to be sensitive, accurate, and quick and offers great prospects for the determination of the 13 mycotoxins studied in natural grasses.

## Conclusions

In this work, a survey of thirteen mycotoxins in natural grass samples from different farms and regions of Spain has been carried out using DMSPE with Fe_3_O_4_@PPy microcomposite as sample treatment. The potential of the magnetic material is demonstrated, as it allows accurate detection of emerging mycotoxin contamination in all samples tested. This multiclass mycotoxin method, compared to others described above, allows the monitoring of the targeted mycotoxins with high sensitivity in such a complex matrix as natural grass for the first time.

Although a disadvantage of the method could be the need for microcomposite synthesis beforehand, reusability studies have shown its capability to be reused up to five times without extraction efficiency losses.

Considering the exciting results described here, we conclude that Fe_3_O_4_@PPy microcomposite has a great potential as adsorbent to be used in DMSPE sample treatment for many challenging matrices as demonstrated with natural grass samples, particularly given the need to strengthen quality control of mycotoxins in the current context of climate change.

## Supplementary information

ESM 1: Supplementary Figures and Tables
