# Supplementary material for: Use of polypyrrole ferrite microparticles and liquid chromatography-mass spectrometry for testing natural grass contamination by multiclass mycotoxins
Source: Mikrochim Acta. 2023 Apr 6;190(5):178. doi: 10.1007/s00604-023-05763-6 (PMC10079724; doi:10.1007/s00604-023-05763-6)
Supplement: Supplementary file 1 — Supplementary Figures and Tables [file 604_2023_5763_MOESM1_ESM.docx]

**Electronic Supplementary Material**

**Use of polypyrrole ferrite microparticles and liquid chromatography - mass spectrometry for testing natural grass contamination by multiclass-mycotoxins**

**M. García-Nicolás^1^, N. Arroyo-Manzanares^1^, N. Campillo^1^, C. Reyes-Palomo^3^,**

**S. Sanz-Fernández^3^, J. Fenoll^2^, V. Rodríguez-Estévez^3^, P. Viñas^1^***

^1^ Department of Analytical Chemistry, Faculty of Chemistry, Regional Campus of International Excellence “Campus Mare Nostrum”, University of Murcia, E-30100 Murcia, Spain.

^2^ Sustainability and Quality Group of Fruit and Vegetable Products, Murcia Institute of Agricultural and Environmental Research and Development, C/ Mayor s/n. La Alberca, 30150, Murcia, Spain.

^3^ Department of Animal Production, UIC ENZOEM, International Agrifood Campus of Excellence (ceiA3), University of Cordoba, Campus de Rabanales, Córdoba, 14071, Spain.

* Corresponding author: pilarvi@um.es


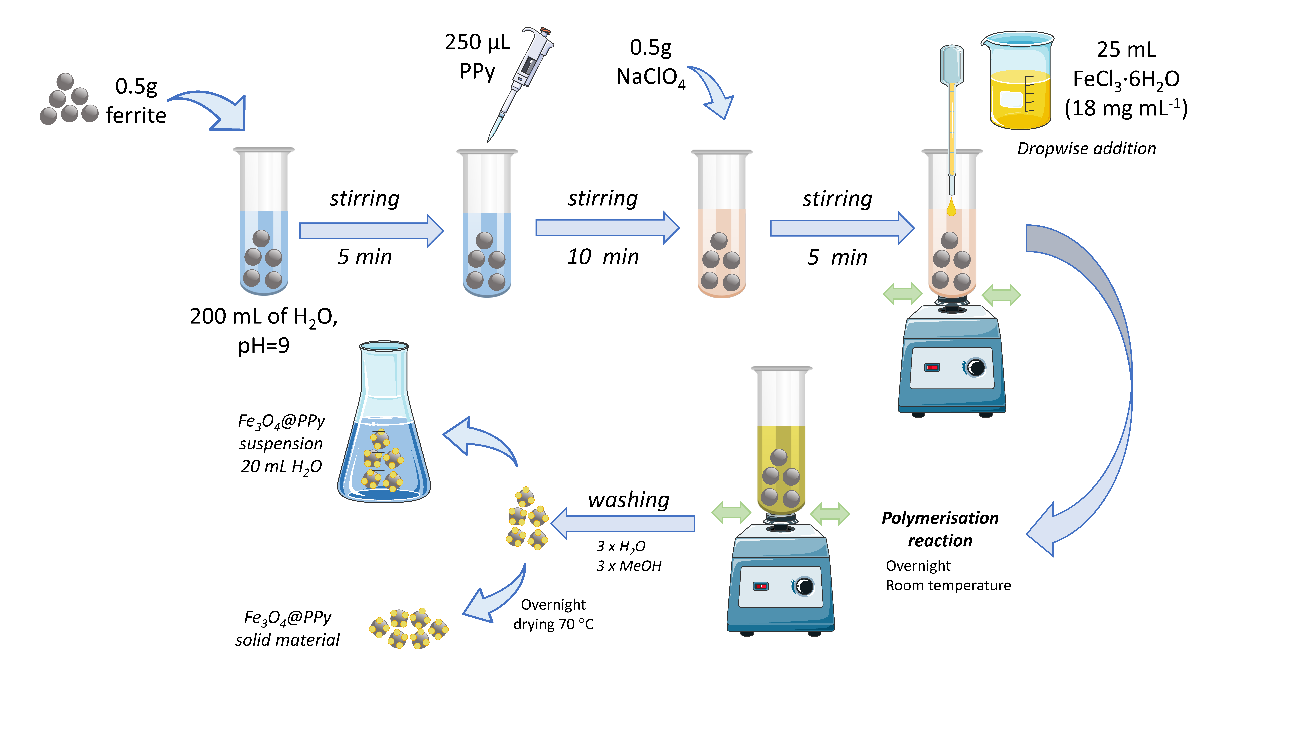


**Figure S1.** Fe_3_O_4_@PPy microcomposite synthesis scheme.

**
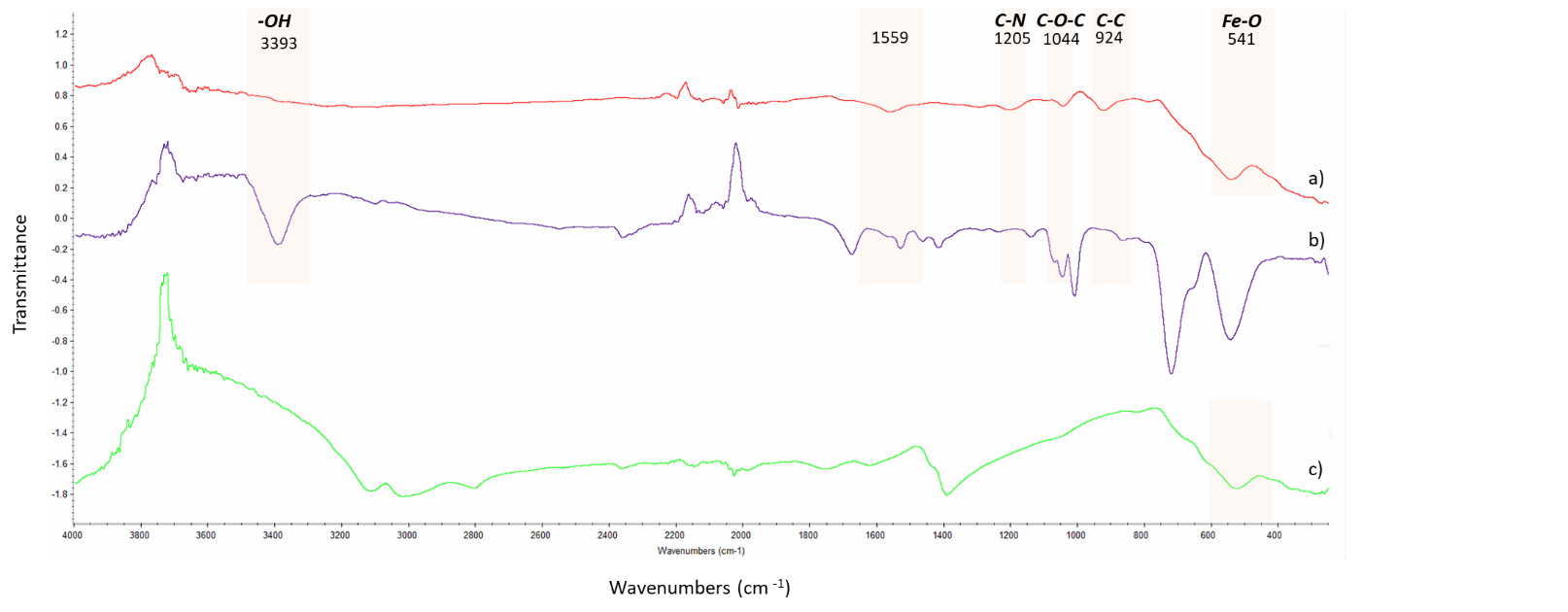
**

**Figure S2.** FTIR spectra of Fe_3_O_4_@PPy microcomposite (a), PPy (b) and Fe_3_O_4_ (c).

**Figure S3.** DLS measurements were performed by adding 5, 15 and 25 mg of solid Fe_3_O_4_@PPy to 2 mL of water.


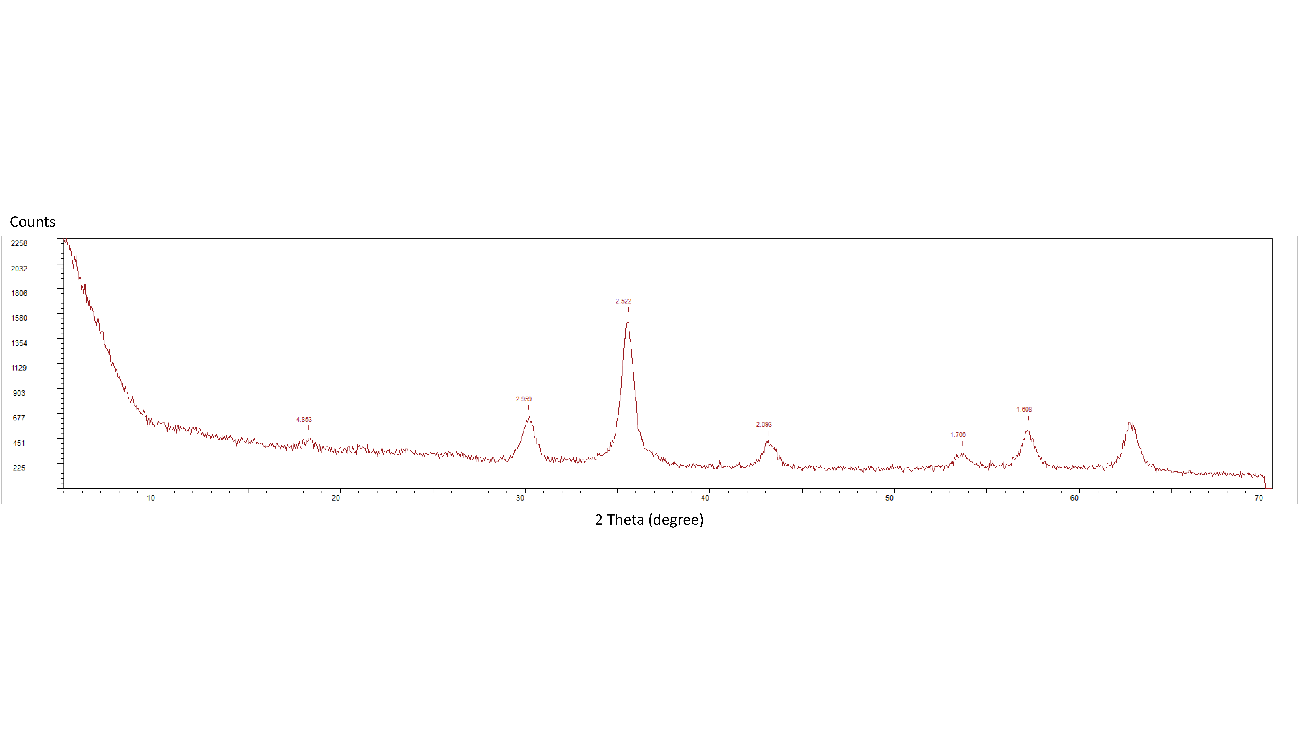


**Figure S4.** X-ray diffraction (XRD) spectrum of Fe_3_O_4_@PPy microcomposite.


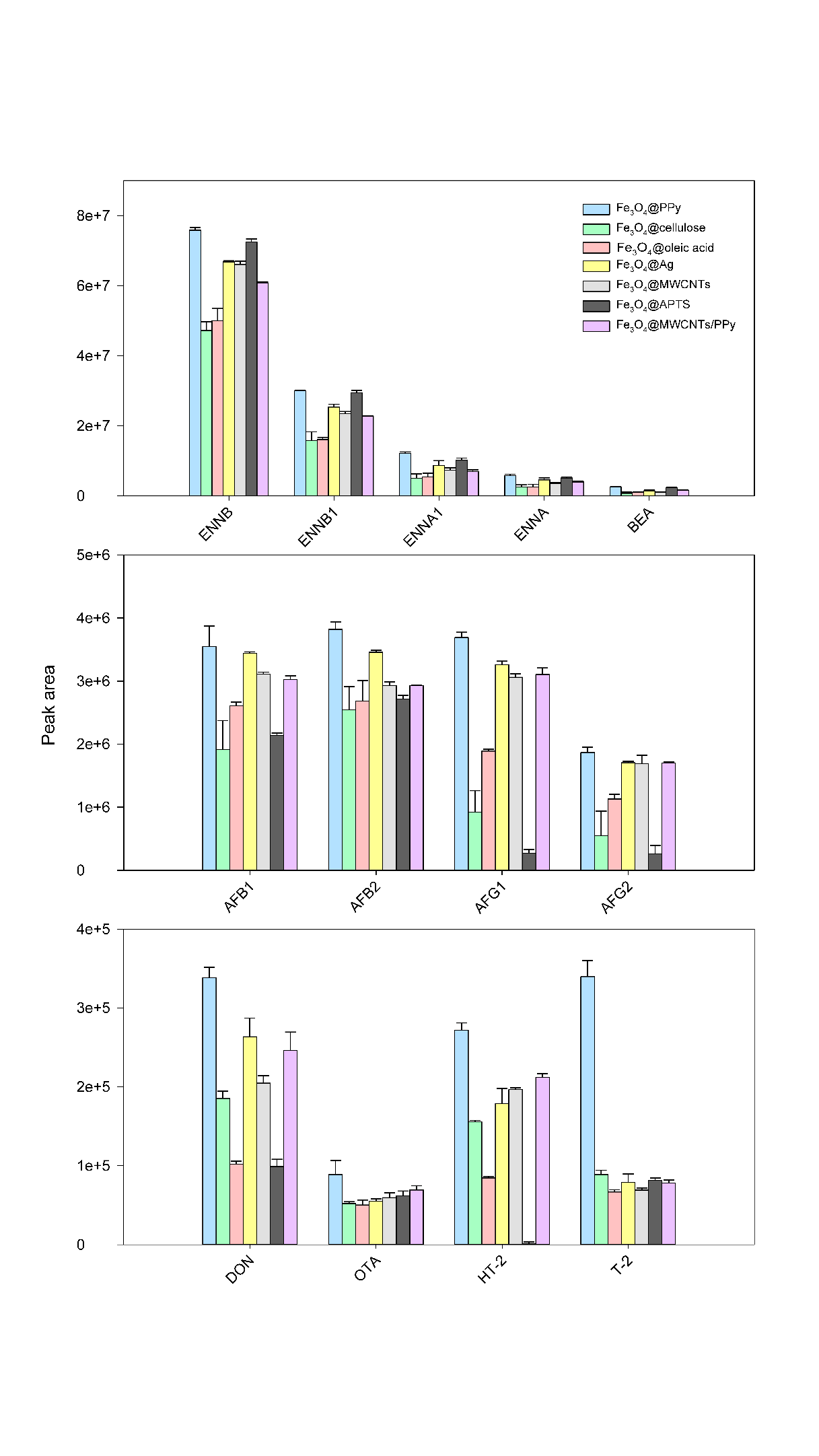


**Figure S5.** Influence of the Fe_3_O_4_@PPy microcomposite type on the sensitivity of the mycotoxin determination (n=3).

**Figure S6.** Influence of the Fe_3_O_4_@PPy microcomposite addition as an aqueous suspension and as solid material (n=3).


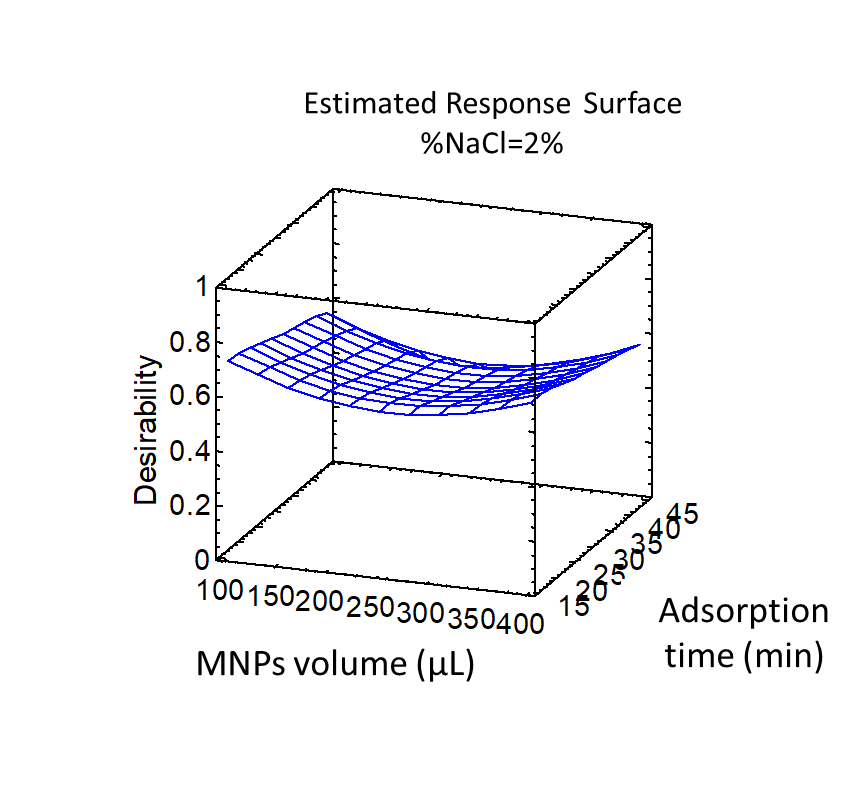


**Figure S7.** Estimated response surface for the study of Fe_3_O_4_@PPy microcomposite suspension volume (100-400 µL), adsorption time (15-45 min) and sodium chloride (0–10% m/v) concentration influence in the DMSPE adsorption step obtained by multiple response optimization (2^3^ + star, face centred, n= 17 runs).


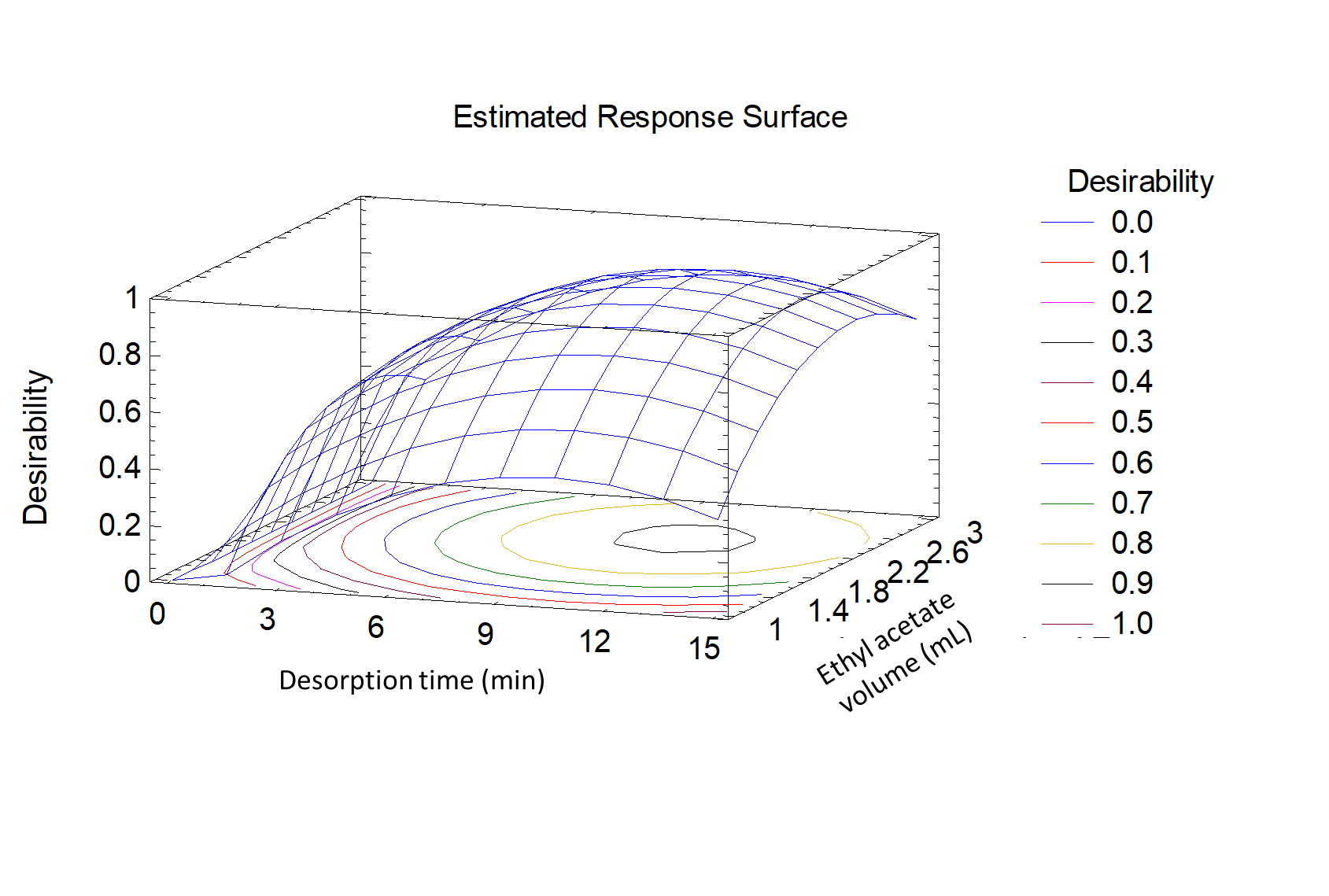


**Figure S8.** Estimated response surface for the study of time and ethyl acetate volume influence (1-15 min, 1-3 mL) in the dispersive magnetic solid-phase extraction (DMSPE) desorption step obtained by multiple response optimization (2^3^ + star, face centred, n= 11 runs).

**DON**

**HT-2**

**AFG_2_**

**AFG_1_**

**AFB_2_**

**AFB_1_**

**T-2**

**OTA**

**ENNB**

**ENNB_1_**

**BEA**

**ENNA_1_**

**ENNA**

**Figure S9.** Chromatogram of 13 mycotoxins analysed using the developed Fe_3_O_4_@PPy microcomposite- based method (10 µg kg^-1^ for the four AFs, OTA, BEA and the four ENNs and 450 µg kg^-1^ for DON, HT-2, and T-2)

**Figure S10.** Box plot obtained after applying one-way analysis of variance test to occurrence concentration data of emergent mycotoxins


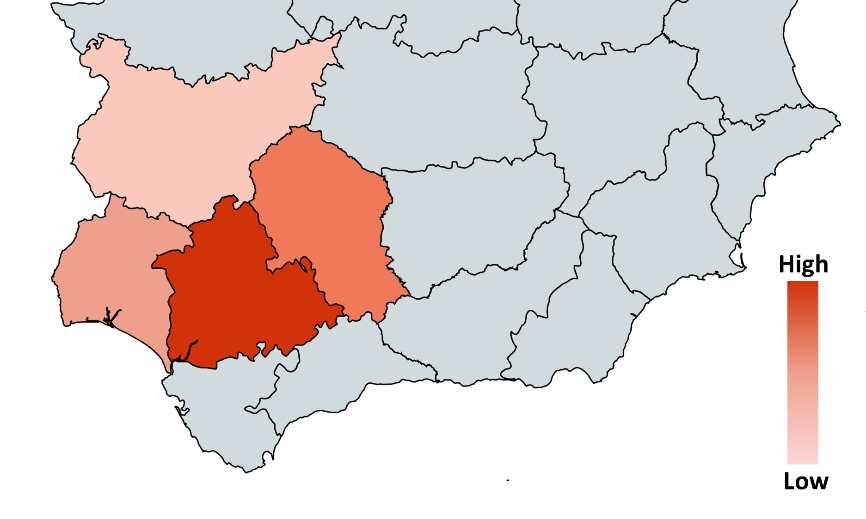


**Figure S11.** Southern Spain map showing significant differences in average contamination from emerging mycotoxin in natural grass from *dehesa* farms.

**Figure S12.** Means plot obtained after applying one-way analysis of variance and least significant difference tests.

**Table S1**. Sample information

| **Sample** | **DM (%)** | **Province** | **Town** | **Farm** | **Collection date** | |
| --- | --- | --- | --- | --- | --- | --- |
| S1 | 84.43 | Sevilla | Constantina | Caños Altos | 07/06/2022 | |
| S2 | 70.59 | Sevilla | Constantina | Caños Altos | 07/06/2022 | |
| S3 | 34.82 | Sevilla | Constantina | Caños Altos | 07/06/2022 | |
| S4 | 68.92 | Sevilla | Constantina | Caños Altos | 07/06/2022 | |
| S5 | 79.25 | Sevilla | Constantina | Caños Altos | 07/06/2022 | |
| S6 | 71.70 | Sevilla | Constantina | Caños Altos | 07/06/2022 | |
| S7 | 52.15 | Sevilla | Constantina | Caños Altos | 07/06/2022 | |
| S8 | 54.07 | Sevilla | Constantina | Caños Altos | 07/06/2022 | |
| S9 | 60.80 | Sevilla | Constantina | Caños Altos | 07/06/2022 | |
| S10 | 62.79 | Sevilla | Cazalla de la Sierra | Coronado | 07/06/2022 | |
| S11 | 75.71 | Sevilla | Cazalla de la Sierra | Coronado | 07/06/2022 | |
| S12 | 73.04 | Sevilla | Cazalla de la Sierra | Coronado | 07/06/2022 | |
| S13 | 75.09 | Sevilla | Cazalla de la Sierra | Coronado | 07/06/2022 | |
| S14 | 65.56 | Sevilla | Cazalla de la Sierra | Coronado | 07/06/2022 | |
| S15 | 73.90 | Sevilla | Cazalla de la Sierra | Coronado | 07/06/2022 | |
| S16 | 97.23 | Sevilla | Cazalla de la Sierra | Coronado | 07/06/2022 | |
| S17 | 82.82 | Sevilla | Cazalla de la Sierra | Coronado | 07/06/2022 | |
| S18 | 80.27 | Sevilla | Cazalla de la Sierra | Coronado | 07/06/2022 | |
| S19 | 91.76 | Sevilla | Cazalla de la Sierra | Coronado | 07/06/2022 | |
| S20 | 57.22 | Sevilla | Cazalla de la Sierra | Coronado | 07/06/2022 | |
| S21 | 90.62 | Sevilla | Cazalla de la Sierra | Coronado | 07/06/2022 | |
| S22 | 87.00 | Sevilla | Cazalla de la Sierra | Coronado | 07/06/2022 | |
| S23 | 65.11 | Huelva | Sta. Olalla del Cala | Fundación Monte Mediterráneo | 01/06/2022 | |
| S24 | 61.57 | Huelva | Sta. Olalla del Cala | Fundación Monte Mediterráneo | 01/06/2022 | |
| S25 | 87.11 | Huelva | Sta. Olalla del Cala | Fundación Monte Mediterráneo | 01/06/2022 | |
| S26 | 48.87 | Huelva | Sta. Olalla del Cala | Fundación Monte Mediterráneo | 01/06/2022 | |
| S27 | 62.92 | Huelva | Sta. Olalla del Cala | Fundación Monte Mediterráneo | 01/06/2022 | |
| S28 | 57.05 | Huelva | Sta. Olalla del Cala | Fundación Monte Mediterráneo | 01/06/2022 | |
| S29 | 91.77 | Huelva | Sta. Olalla del Cala | Fundación Monte Mediterráneo | 01/06/2022 | |
| S30 | 65.11 | Huelva | Sta. Olalla del Cala | Fundación Monte Mediterráneo | 01/06/2022 | |
| S31 | 50.57 | Huelva | Sta. Olalla del Cala | Fundación Monte Mediterráneo | 01/06/2022 | |
| S32 | 53.65 | Huelva | Sta. Olalla del Cala | Fundación Monte Mediterráneo | 01/06/2022 | |
| S33 | 63.21 | Huelva | Sta. Olalla del Cala | Fundación Monte Mediterráneo | 01/06/2022 | |
| S34 | 74.63 | Huelva | Sta. Olalla del Cala | Fundación Monte Mediterráneo | 01/06/2022 | |
| S35 | 68.71 | Huelva | Sta. Olalla del Cala | Fundación Monte Mediterráneo | 01/06/2022 | |
| S36 | 85.39 | Huelva | Sta. Olalla del Cala | Fundación Monte Mediterráneo | 01/06/2022 | |
| S37 | 43.78 | Huelva | Sta. Olalla del Cala | Fundación Monte Mediterráneo | 01/06/2022 | |
| S38 | 65.45 | Sevilla | Cazalla de la Sierra | Lagarón | 07/06/2022 | |
| S39 | 93.03 | Sevilla | Cazalla de la Sierra | Lagarón | 07/06/2022 | |
| S40 | 85.00 | Sevilla | Cazalla de la Sierra | Lagarón | 07/06/2022 | |
| S41 | 87.10 | Sevilla | Cazalla de la Sierra | Lagarón | 07/06/2022 | |
| S42 | 63.13 | Sevilla | Cazalla de la Sierra | Lagarón | 07/06/2022 | |
| S43 | 87.47 | Sevilla | Cazalla de la Sierra | Lagarón | 07/06/2022 | |
| S44 | 84.01 | Sevilla | Cazalla de la Sierra | Lagarón | 07/06/2022 | |
| S45 | 90.47 | Sevilla | Cazalla de la Sierra | Lagarón | 07/06/2022 | |
| S46 | 86.60 | Sevilla | Cazalla de la Sierra | Lagarón | 07/06/2022 | |
| S47 | 39.89 | Córdoba | Pozoblanco | La Loma | 23/05/2022 | |
| S48 | 55.24 | Córdoba | Pozoblanco | La Loma | 23/05/2022 | |
| S49 | 75.33 | Córdoba | Pozoblanco | La Loma | 23/05/2022 | |
| S50 | 64.61 | Córdoba | Pozoblanco | La Loma | 23/05/2022 | |
| S51 | 79.88 | Córdoba | Pozoblanco | La Loma | 23/05/2022 | |
| S52 | 72.36 | Córdoba | Pozoblanco | La Loma | 23/05/2022 | |
| S53 | 57.50 | Córdoba | Pozoblanco | La Loma | 23/05/2022 | |
| S54 | 51.16 | Córdoba | Pozoblanco | La Loma | 23/05/2022 | |
| S55 | 72.41 | Córdoba | Pozoblanco | La Loma | 23/05/2022 | |
| S56 | 48.48 | Córdoba | Pozoblanco | Navalpozuelo | 23/05/2022 | |
| S57 | 43.69 | Córdoba | Pozoblanco | Navalpozuelo | 23/05/2022 | |
| S58 | 48.77 | Córdoba | Pozoblanco | Navalpozuelo | 23/05/2022 | |
| S59 | 53.04 | Córdoba | Pozoblanco | Navalpozuelo | 23/05/2022 | |
| S60 | 30.12 | Córdoba | Pozoblanco | Navalpozuelo | 23/05/2022 | |
| S61 | 60.01 | Córdoba | Pozoblanco | Navalpozuelo | 23/05/2022 | |
| S62 | 37.92 | Córdoba | Pozoblanco | Navalpozuelo | 23/05/2022 | |
| S63 | 32.75 | Córdoba | Pozoblanco | Navalpozuelo | 23/05/2022 | |
| S64 | 32.01 | Córdoba | Pozoblanco | El Palomar | 23/05/2022 | |
| S65 | 46.17 | Córdoba | Pozoblanco | El Palomar | 23/05/2022 | |
| S66 | 58.04 | Córdoba | Pozoblanco | El Palomar | 23/05/2022 | |
| S67 | 54.28 | Córdoba | Pozoblanco | El Palomar | 23/05/2022 | |
| S68 | 73.07 | Córdoba | Pozoblanco | El Palomar | 23/05/2022 | |
| S69 | 32.01 | Córdoba | Pozoblanco | El Palomar | 23/05/2022 | |
| S70 | 54.60 | Córdoba | Pozoblanco | El Palomar | 23/05/2022 | |
| S71 | 54.60 | Córdoba | Pozoblanco | El Palomar | 23/05/2022 | |
| S72 | 57.15 | Córdoba | Pozoblanco | El Palomar | 23/05/2022 | |
| S73 | 58.52 | Córdoba | Pozoblanco | El Palomar | 23/05/2022 | |
| S74 | 63.03 | Córdoba | Pozoblanco | El Palomar | 23/05/2022 | |
| S75 | 60.96 | Córdoba | Pozoblanco | El Palomar | 23/05/2022 | |
| S76 | 80.15 | Badajoz | Calamonte | La Rinconada | 01/06/2022 | |
| S77 | 70.53 | Badajoz | Calamonte | La Rinconada | 01/06/2022 | |
| S78 | 78.88 | Badajoz | Calamonte | La Rinconada | 01/06/2022 | |
| S79 | 83.03 | Badajoz | Calamonte | La Rinconada | 01/06/2022 | |
| S80 | 85.61 | Badajoz | Calamonte | La Rinconada | 01/06/2022 | |
| S81 | 74.76 | Badajoz | Calamonte | La Rinconada | 01/06/2022 | |
| S82 | 84.43 | Badajoz | Calamonte | La Rinconada | 01/06/2022 | |
| S83 | 83.47 | Badajoz | Calamonte | La Rinconada | 01/06/2022 | |
| DM, dry matter | | | | | |  |

**Table S2**. LC-QqQ-MS/MS parameters for the mycotoxin determination.

| **Analyte** | **RT^a^ (min)** | **Molecular formula** | **Precursor ion (*m/z*)** | **Molecular ion** | **Product ion^b^** | **Fragmentor (V)** | **CE^c^ (V)** |
| --- | --- | --- | --- | --- | --- | --- | --- |
| DON | 4.56 | C_15_H_20_O_6_ | 297.00 | [M+H]^+^ | 249 (Q) | 120 | 4 |
|  |  |  |  |  | 77 (q) | 120 | 80 |
| AFG_2_ | 10.35 | C_17_H_14_O_7_ | 331.00 | [M+H]^+^ | 313 (Q) | 160 | 23 |
|  |  |  |  |  | 189 (q) | 160 | 45 |
| AFG_1_ | 11.17 | C_17_H_12_O_7_ | 329.00 | [M+H]^+^ | 243 (Q) | 160 | 25 |
|  |  |  |  |  | 200 (q) | 160 | 45 |
| AFB_2_ | 11.92 | C_17_H_14_O_6_ | 315.00 | [M+H]^+^ | 259 (Q) | 160 | 30 |
|  |  |  |  |  | 287 (q) | 160 | 25 |
| AFB_1_ | 12.61 | C_17_H_12_O_6_ | 313.00 | [M+H]^+^ | 241(Q) | 160 | 40 |
|  |  |  |  |  | 128 (q) | 160 | 85 |
| HT-2 | 15.16 | C_22_H_32_O_8_ | 442.00 | [M+NH_4_]^+^ | 215 (Q) | 120 | 10 |
|  |  |  |  |  | 263 (q) | 120 | 7 |
| T-2 | 16.53 | C_24_H_34_O_9_ | 484.00 | [M+NH_4_]^+^ | 185 (Q) | 120 | 20 |
|  |  |  |  |  | 245 (q) | 120 | 7 |
| OTA | 18.03 | C_20_H_18_ClNO_6_ | 404.00 | [M+H]^+^ | 239 (Q) | 124 | 25 |
|  |  |  |  |  | 221 (q) | 124 | 36 |
| ENNB | 21.78 | C_33_H_57_N_3_O_9_ | 657.50 | [M+NH_4_]^+^ | 196 (Q) | 130 | 30 |
|  |  |  |  |  | 214 (q) | 130 | 30 |
| BEA | 22.10 | C_45_H_57_N_3_O_9_ | 801.50 | [M+NH_4_]^+^ | 244 (Q) | 130 | 30 |
|  |  |  |  |  | 262 (q) | 130 | 30 |
| ENNB_1_ | 22.21 | C_34_H_59_N_3_O_9_ | 671.60 | [M+NH_4_]^+^ | 196 (Q) | 130 | 30 |
|  |  |  |  |  | 214 (q) | 130 | 40 |
| ENNA_1_ | 22.62 | C_35_H_61_N_3_O_9_ | 685.60 | [M+NH_4_]^+^ | 210 (Q) | 130 | 30 |
|  |  |  |  |  | 228 (q) | 130 | 30 |
| ENNA | 22.97 | C_36_H_63_N_3_O_6_ | 699.60 | [M+NH_4_]^+^ | 210 (Q) | 130 | 30 |
|  |  |  |  |  | 228 (q) | 130 | 30 |
| ^a^ Retention time ^b^ Q, quantification ion; q, qualifier ion ^c^ Collision energy | | | | | | | |

**Table S3**. Mycotoxin metabolites monitored using a non-targeted approach.

| **Compound** | **Abbreviation** | **Molecular Formula** | **Monoisotopic mass (Da)** |
| --- | --- | --- | --- |
| 10-Deepoxy-deoxynivalenol-1-sulfonate | 10-DOM-1-sulfonate | C_15_H_21_O_8_S | 361.0957 |
| Deepoxy deoxynivalenol | DOM-1 | C_15_H_20_O_5_ | 280.1311 |
| Iso-deepoxydeoxynivalenol | Iso-DOM | C_15_H_20_O_5_ | 280.1311 |
| Deepoxy-deoxynivalenol-15/3-glucuronide | DOM-15/3-glucuronide | C_21_H_28_O_11_ | 456.1632 |
| Deoxynivalenol-15/3-sulfate | DON-15/3-sulfate | C_15_H_19_O_9_S | 375.075 |
| Deoxynivalenol sulfonate 1/2/3 | DON S_1_/S_2_/S_3_ | C_15_H_19_O_9_S | 375.075 |
| Deoxynivalenol-15/3-glucuronide | DON-15/3-glucuronide | C_21_H_28_O_12_ | 472.1581 |
| Deoxynivalenol-8,15-hemiketal-8-glucuronide | DON-8,15-hemiketal-8-glucuronide | C_21_H_29_O_13_ | 489.1608 |
| Iso-deepoxy-deoxynivalenol-15/3/8-glucuronide | Iso-DOM-15/3/8-glucuronide | C_21_H_30_O_11_ | 458.1788 |
| Iso-deoxynivalenol | Iso-DON | C_15_H_20_O_6_ | 296.126 |
| Iso-deoxynivalenol-15/3/8-glucuronide | Iso-DON-15/3/8-glucuronide | C_21_H_30_O_11_ | 458.1788 |
| 15-deacetylneosolaniol | 15-deAc-NEO | C_17_H_24_O_7_ | 340.1522 |
| 15-deacetyl-T-2 | 15-deacetyl-T-2 | C_22_H_32_O_8_ | 424.2097 |
| 3’,7-dihydroxy-HT-2 | 3’-7-diOH-HT-2 | C_22_H_32_O_10_ | 456.1995 |
| 3’,7-dihydroxy-HT-2 (isomer) | 3’-7-diOH-HT-2 (isomer) | C_22_H_32_O_10_ | 456.1995 |
| 3’,7-dihydroxy-T-2 | 3’-7-diOH-T-2 | C_24_H_34_O_11_ | 498.2101 |
| 3’-hydroxy-9-hydroxy-T-2 | 3’-OH-9-OH-T-2 | C_24_H_36_O_11_ | 500.2258 |
| 3’-hydroxy-T-2-glucoside | 3’-OH-T-2-glucoside | C_30_H_44_O_15_ | 644.268 |
| 3’,4’-dihydroxy-T-2 | 3’,4’-di-OH-T-2 | C_22_H_32_O_8_ | 424.2087 |
| 3’,4’-dihydroxy-T-2 (isomer) | 3’,4’-di-OH-T-2 (isomer) | C_22_H_32_O_8_ | 424.2087 |
| 3-hydroxy-15-deacetyl-T-2 | 3-OH-15-deacetyl-T-2 | C_22_H_32_O_9_ | 440.2046 |
| 3-hydroxy-HT-2 (T-2 triol) | 3’-OH-HT-2 | C_20_H_30_O_7_ | 382.1992 |
| 3-hydroxy-HT-2-3-sulfate | 3’-OH-HT-2-3-SO_3_H | C_24_H_39_O_11_S | 535.2213 |
| 3-hydroxy-T-2 | 3’-OH-T-2 | C_24_H_34_O_10_ | 482.2152 |
| 3-hydroxy-T-2-3-sulfate | 3’-OH-T-2-3-SO_3_H | C_26_H_41_O_12_S | 577.2319 |
| 4’-carboxyl-3’-hydroxy-T-2 | 4’-COOH-3’-OH-T-2 | C_17_H_24_O_7_ | 340.1522 |
| 4’-carboxyl-3’-hydroxy-T-2 (isomer) | 4’-COOH-3’-OH-T-2 (isomer) | C_17_H_24_O_7_ | 340.1522 |
| 4’-carboxyl-HT-2 | 4’-COOH-HT-2 | C_22_H_32_O_8_ | 424.2097 |
| 4’-carboxyl-HT-2 (isomer) | 4’-COOH-HT-2 (isomer) | C_22_H_32_O_8_ | 424.2097 |
| 4’-carboxyl-T-2 | 4’-COOH-T-2 | C_17_H_24_O_7_ | 340.1522 |
| 4’-carboxyl-T-2 (isomer) | 4’-COOH-T-2 (isomer) | C_17_H_24_O_7_ | 340.1522 |
| 4’-hydroxy-T-2-glucoside | 4’-OH-T-2-glucoside | C_30_H_44_O_15_ | 644.268 |
| 4’-hydroxy-T-2-glucoside (isomer) | 4’-OH-T-2-glucoside (isomer) | C_30_H_44_O_15_ | 644.268 |
| 4’,4’-dihydroxy-T-2 | 4’,4’-di-OH-T-2 | C_22_H_32_O_8_ | 424.2097 |
| 4-deacetylneosolaniol | 4-deAc-NEO | C_17_H_24_O_7_ | 340.1522 |
| 4-hydroxy-HT-2 | 4’-OH-HT-2 | C_22_H_32_O_9_ | 440.2046 |
| 4-hydroxy-HT-2 (isomer) | 4’-OH-HT-2 (isomer) | C_22_H_32_O_9_ | 440.2046 |
| 7-hydroxy-HT-2 | 7-OH-HT-2 | C_28_H_42_O_14_ | 602.2575 |
| 7-hydroxy-HT-2 (isomer) | 7-OH-HT-2 (isomer) | C_28_H_42_O_14_ | 602.2575 |
| 9-hydroxyl-T-2 | 9-OH-T-2 | C_24_H_36_O_10_ | 484.2308 |
| De-epoxy-3’,7-dihydroxy-HT-2 | De-epoxy-3’,7-diOH-HT-2 | C_22_H_32_O_9_ | 440.2046 |
| De-epoxy-3’-hydroxy-HT-2 | De-epoxy-3’-OH-HT-2 | C_22_H_32_O_8_ | 424.2097 |
| De-epoxy-3’-hydroxy-T-2 triol | De-epoxy-3’-OH-T-2 triol | C_24_H_34_O_9_ | 466.2203 |
| De-epoxy-HT-2 | De-epoxy-HT-2 | C_22_H_32_O_7_ | 408.2148 |
| HT-2-3/2-4-glucuronide | HT-2-3/2-4-glucuronide | C_28_H_40_O_14_ | 600.2418 |
| HT-2-glucoside | HT-2-glucoside | C_28_H_42_O_13_ | 586.2625 |
| Neosolaniol | NEO | C_19_H_26_O_8_ | 382.1628 |
| Neosolaniol-3-glucoside | NEO-3-glucoside | C_25_H_36_O_13_ | 544.2156 |
| T-2 tetraol | T-2 tetraol | C_15_H_22_O_6_ | 298.1416 |
| T-2-3-glucuronide | T-2-3-glucuronide | C_30_H_42_O_15_ | 642.2524 |
| 10/13/15/2/3/4/5/6/8/9-hydroxy-zearalenone | 10/13/15/2/3/4/5/6/8/9-OH-ZEN | C_18_H_22_O_6_ | 334.1416 |
| 4/5/6/8/9-hydroxy-zearalenone (isomer) | 4/5/6/8/9-OH-ZEN (isomer) | C_18_H_22_O_6_ | 334.1416 |
| De-epoxy-zearalenone | De-epoxy-ZEN | C_18_H_22_O_6_ | 334.1416 |
| De-epoxy-zearalenone (isomer) | De-epoxy-ZEN (isomer) | C_18_H_22_O_6_ | 334.1416 |
| Hydroxy-zearalenone-glucuronide | OH-ZEN-glucuronide | C_24_H_30_O_12_ | 510.1737 |
| Zearalenone-14,16/14,2-di-glucuronide | ZEN-14,16/14,2-di-glucuronide | C_30_H_38_O_17_ | 670.2109 |
| Zearalenone-14/16-glucuronide | ZEN-14/16-glucuronide | C_24_H_30_O_11_ | 494.1788 |
| Zearalenone-14-sulphate | ZEN-14-SO_3_H | C_18_H_22_SO_8_ | 398.1035 |
| α-zearalenol/β-zearalenol | α-ZEL/β-ZEL | C_18_H_24_O_5_ | 320.1624 |
| α-zearalenol/β-zearalenol-14-glucuronide | α-ZEL/β-ZEL-14-glucuronide | C_24_H_32_O_11_ | 496.1945 |
| α-zearalenol/β-zearalenol-16-glucuronide | α-ZEL/β-ZEL-16-glucuronide | C_24_H_32_O_11_ | 496.1945 |
| α-zearalenol/β-zearalenol-14-sulphate | α-ZEL/β-ZEL-14- SO_3_H | C_18_H_24_SO_8_ | 400.1192 |
| Aflatoxin B_1_ 8,9-dihydrodiol | AFB_1_ 8,9-dihydrodiol | C_17_H_14_O_8_ | 346.0639 |
| Aflatoxin B_1_ glutathione conjugate | AFB_1_-GSH | C_27_H_29_N_3_O_13_S | 635.1421 |
| Aflatoxin B_1_ 8,9-epoxide | AFBO | C_17_H_12_O_7_ | 328.0583 |
| Aflatoxin B_1_-lysine | AFB_1_-lysine | C_23_H_25_N_2_O_8_ | 457.1611 |
| Aflatoxin B-N7-guanine | AFB-N7-guanine | C_22_H_16_N_5_O_7_ | 462.105 |
| Aflatoxin M_1_ 8,9-dihydrodiol | AFM_1_ 8,9-dihydrodiol | C_17_H_14_O_8_ | 346.0689 |
| Aflatoxin B_2a_ | AFB_2a_ | C_17_H_14_O_7_ | 330.0739 |
| Aflatoxin G_2a_ | AFG_2a_ | C_17_H_14_O_8_ | 346.0689 |
| Aflatoxin GM_1_ | AFGM_1_ | C_17_H_12_O_8_ | 344.0532 |
| Aflatoxin M_4_ | AFM_4_ | C_17_H_12_O_7_ | 328.0583 |
| Aflatoxin P_2_ | AFP_2_ | C_16_H_12_O_6_ | 300.0634 |
| Aflatoxin M_2_ | AFM_2_ | C_17_H_14_O_7_ | 331.08 |
| Aflatoxin P_1_ | AFP_1_ | C_16_H_10_O_6_ | 299.05 |
| Aflatoxin M_2a_ | AFM_2a_ | C_17_H_14_O_8_ | 346.0689 |
| Aflatoxin Q_1_ | AFQ_1_ | C_17_H_12_O_7_ | 329.06 |
| Aflatoxin Q_2a_ | AFQ_2a_ | C_17_H_14_O_8_ | 346.0689 |
| Aflatoxicol | AFL | C_17_H_14_O_6_ | 314.079 |
| Aflatoxicol B | AFLB | C_17_H_14_O_6_ | 314.079 |
| Aflatoxicol H_1_ | AFLH_1_ | C_17_H_14_O_7_ | 330.0739 |
| Aflatoxicol M_1_ | AFLM_1_ | C_17_H_14_O_7_ | 330.0739 |
| Beauvericin A/F | BEA A/F | C_46_H_59_N_3_O_9_ | 797.4251 |
| Allobeauvericin A | ALLOBEA A | C_46_H_59_N_3_O_9_ | 797.4251 |
| Beauvericin B | BEA B | C_47_H_61_N_3_O_9_ | 811.4408 |
| Allobeauvericin B | ALLOBEA B | C_47_H_61_N_3_O_9_ | 811.4408 |
| Beauvericin C | BEA C | C_48_H_63_N_3_O_9_ | 825.4564 |
| Allobeauvericin C | ALLOBEA C | C_48_H_63_N_3_O_9_ | 825.4564 |
| Beauvericin D | BEA D | C_44_H_55_N_3_O_9_ | 769.3938 |
| Beauvericin E | BEA E | C_41_H_57_N_3_O_9_ | 735.4095 |
| Beauvericin G_1_ | BEA G_1_ | C_44_H_55_N_3_O_9_ | 769.3938 |
| Beauvericin G_2_ | BEA G_2_ | C_43_H_53_N_3_O_9_ | 755.3782 |
| Beauvericin G_3_ | BEA G_3_ | C_42_H_51_N_3_O_9_ | 741.3625 |
| Beauvericin H_1_ | BEA H_1_ | C_45_H_56_FN_3_O_9_ | 801.4001 |
| Beauvericin H_2_ | BEA H_2_ | C_45_H_55_F_2_N_3_O_9_ | 819.3906 |
| Beauvericin H_3_ | BEA H_3_ | C_45_H_54_F_3_N_3_O_9_ | 837.3812 |
| Beauvericin J | BEA J | C_45_H_57_N_3_O_10_ | 799.4044 |
| Beauvericin K | BEA K | C_45_H_57_N_3_O_11_ | 815.3993 |
| Beauvericin L | BEA L | C_45_H_57_N_3_O_12_ | 831.3942 |
| Beauvenniatin A | BEAE A | C_41_H_57_N_3_O_9_ | 735.4095 |
| Beauvenniatin B | BEAE B | C_37_H_57_N_3_O_9_ | 687.4095 |
| Beauvenniatin G_1_/G_2_/G_3_ | BEAE G_1_/G_2_/G_3_ | C_39_H_61_N_3_O_9_ | 715.4408 |
| Beauvenniatin L | BEAE L | C_42_H_59_N_3_O_9_ | 749.4251 |
| Enniatin F/MK 1688 | ENN F/MK 1688 | C_36_H_63_N_3_O_9_ | 681.4564 |
| Enniatin E/I | ENN E/I | C_35_H_61_N_3_O_9_ | 667.4408 |
| Enniatin A_2_/A_3_ | ENN A_2_/A_3_ | C_36_H_63_N_3_O_9_ | 681.4564 |
| Enniatin B_4_/D/H | ENN B_4_/D/H | C_34_H_59_N_3_O_9_ | 653.4251 |
| Enniatin B_2_/J_2_/J_3_/K_1_ | ENN B_2_/J_2_/J_3_/K_1_ | C_32_H_55_N_3_O_9_ | 625.3938 |
| Enniatin B_3_/J_1_ | ENN B_3_/J_1_ | C_31_H_53_N_3_O_9_ | 613.3782 |
| Enniatin O_1_/O_2_/O_3_ | ENN O_1_/O_2_/O_3_ | C_35_H_61_N_3_O_9_ | 667.4408 |
| Enniatin Q | ENN Q | C_36_H_63_N_3_O_9_ | 681.4564 |
| Enniatin M_1_/M_2_/S | ENN M_1_/M_2_/S | C_35_H_61_N_3_O_10_ | 683.4357 |
| Enniatin P_1_ | ENN P_1_ | C_33_H_57_N_3_O_10_ | 655.4044 |
| Enniatin P_2_/R | ENN P_2_/R | C_34_H_59_N_3_O_10_ | 669.42 |
| Enniatin T | ENN T | C_36_H_63_N_3_O_12_ | 730.449 |
| Enniatin U | ENN U | C_35_H_61_N_3_O_11_ | 700.4384 |
| Enniatin V | ENN V | C_36_H_63_N_3_O_11_ | 714.4541 |

| **Table S4**. An overview on recently reported nano/micromaterial-based chromatographic methods for the determination of the mycotoxins. | | | | | | | | | |
| --- | --- | --- | --- | --- | --- | --- | --- | --- | --- |
| **NPs type** | **Sample** | **Mycotoxins** | **Determination technique** | **Sorbent amount** | **Sample preparation time** | **Recovery (%)** | **RSD (%)** | **LOD** | **Ref** |
| Fe_3_O_4_/COF-TpBD | Maize | AFB_1_, AFB_2_, AFG_1_, AFG_2_, OTA, OTB, ENNB, ENNA_1_, ENNA, ENNB_1_ | HPLC-MS/MS | 5 mg | 32 min | 73.8-103.1 | 2.1-8.5 | 0.02 (ENNA) – 1.67 (AFG_2_) µg kg^-1^ | Wei *et al.* 2023 |
| PEG-MWCNTs-MNP | Liquid milk | AFB_1_, AFB_2_, AFG_1_, AFG_2_, AFM_1_, AFM_2_, OTA, ZEA, ZAN, α-ZAL, β-ZAL, α-ZOL, β-ZOL | UHPLC-Q-Exactive HRMS | 10 mg | 43 min | 81.8-106.4 | 2.1-11.7 | 0.005 (AFM_1_, AFM_2_) – 0.05 (OTA, ZAN, α-ZAL, β-ZAL) µg kg^-1^ | Zhao *et al.* 2020 |
| Fe_3_O_4_-MWCNTs-NH_2_ | Wheat flour | AFB_1_, ZEA | HPLC-DAD | 6 mg | 1h 10 min | 88.8-96.0 | 1.6-6.6 | 0.15 (AFB_1_) – 0.24 (ZEA) ng g^-1^ | Li *et al.* 2018 |
| Fe_3_O_4_@PDA/MIL-101(Cr) | Licorice | AFB_1_, AFG_1_, STE, ZEA, OTA | UHPLC-MS/MS | 20 mg | 1h 7 min | 78.5-116.3 | 6.0-11.2 | 0.01 (OTA, ZEA) – 0.09 (STE) µg kg^-1^ | Tang *et al.*2022 |
| Fe_3_O_4_@nSiO_2_@mSiO_2_ | Vegetable oil | FB_1_, ZEA, OTA | UPLC-MS/MS | 5 mg | 15 min | 89.4-97.1 | 2.9-5.7 | 0.08 (OTA) – 1.03 (ZEA) µg kg^-1^ | Zhao *et al.*2017 |
| PDA@ Fe_3_O_4_-MWCNTs | Edible vegetable oils | AFB_1_, AFB_2_, AFG_1_, AFG_2_, OTA, OTB | HPLC-FLD | 50 mg | 43 min | 70.5-89.3 | 1.2-6.3 | 0.2 (AFs)-0.5 (OTA, OTB) µg kg^-1^ | Xu *et al.* 2021 |
| ILM/ZIF-8 | Milk samples | AFB_1_, AFB_2_, AFG_1_, AFG_2_ | UHPLC-MS/MS | 90 mg | 16 min | 80.2 – 102.5 | 3.5-7.7 | 2.3 (AFB_1_) – 8.1 (AFG_1_) ng L^-1^ | Gao *et al.* 2019 |
| Fe_3_O_4_@PDA MIPs | Rice, wine | OTA, OTB, OTC | HPLC-FLD | 15 mg | - | 71.0-88.5 | 2.3-3.8 | 1.8 (OTA)- 18 (OTB) pg·mL^−1^, | Hu *et al.* 2018 |
| Fe_3_O_4_@PPy | Natural grass | DON, AFG_2_, AFG_1_, AFB_2_, AFB_1_, HT-2, T-2, OTA, ENNB, ENNA_1_, ENNA, ENNB_1_, BEA | LC-QqQ-MS/MS | 400 µL  (390 mg) | 25 min | 82.0-110.0 | 1.5-10.2 | 0.02 (ENNA, A_1_ and B) – 27 (DON) µg kg^-1^ | This work |
| Fe_3_O_4_/COF-TpBD: core-shell structured magnetic covalent organic framework.  PEG-MWCNTs-MNP: PEGylated multi-walled carbon nanotubes magnetic nanoparticles.  Fe_3_O_4_-MWCNTs-NH_2_: ferrite nanoparticle-filled amino-modified multi-walled carbon nanotubes.  Fe_3_O_4_@PDA/MIL-101(Cr): ferrite polydopamine MIL-101(Cr) nanomaterial.  Fe_3_O_4_@nSiO_2_@mSiO_2_: magnetic nanoparticles coated with double layers of silicon dioxide.  PDA@ Fe_3_O_4_-MWCNTs: polydopamine-coated magnetic multi-walled carbon nanotubes.  ILM/ZIF-8: ionic liquid magnetic zeolite imidazolate framework-8.  Fe_3_O_4_@PDA MIPs: molecularly imprinted polymer deposited on the surface of magnetite (ferroferric oxide) nanoparticles.  Fe_3_O_4_@PPy: polypyrrol coated magnetic microcomposite.  Mycotoxins: aflatoxin B1 (AFB1), aflatoxin B2 (AFB2), aflatoxin G1 (AFG1), aflatoxin G2 (AFG2), ochratoxin A (OTA), ochratoxin B (OTB), aflatoxin M1 (AFM1), aflatoxin M2 (AFM2), ochratoxin A (OTA), zearalenone (ZEA), zearalanone (ZAN), α-zeralanol (α-ZAL), β-zeralanol (β-ZAL), α-zeralenol (α-ZOL), and β-zeralenol (β-ZOL) ochratoxin B ( OTB), sterigmatocystin (STE) | | | | | | | | | |

**Fe_3_O_4_@PPy microcomposite synthesis**

After obtaining the ferrite (Fe_3_O_4_) magnetic core, the Fe_3_O_4_@PPy MNPs was generated. For this purpose, an amount of 0.5 g of the dried Fe_3_O_4_ was dissolved in 200 mL of deionized water under continuous stirring at pH 9 for 5 min. Then, the mixture was stirred for 10 min after the addition of 0.25 mL of pyrrole. Subsequently, 0.5 g of sodium perchlorate was added and stirred for 5 min, and then 25 mL of 18 mg mL^-1^ FeCl_3_·6H_2_O solution was added dropwise to the mixture while being stirred. Polymerisation reaction was allowed to proceed by leaving the preparation under orbital shaking overnight at room temperature. Finally, a water and EtOH washing of the microcomposite was performed for three times until neutral pH.

The microcomposite was prepared as suspension or as solid material. A 20 mL volume of suspension was prepared in a concentration of 976 mg mL^-1^ Fe_3_O_4_@PPy and the solid material was obtained by drying overnight at 70 ºC the previously synthetised Fe_3_O_4_@PPy.

**Fe_3_O_4_@cellulose nanocomposite synthesis**

The magnetic Fe_3_O_4_@cellulose nanocomposite material was synthesized following the procedure described by García-Nicolás et al. 2021. In the first step, 2 g of microcrystalline cellulose was dissolved in 100 mL of a 2% solution of 8:6.5:8:77.5 NaOH:thiourea:urea:H_2_O and stirred for 30 min at 4 °C to form the homogenous cellulose solution. Then 0.1 mol of Na_2_CO_3_ and 0.1 mol of NaOH contained in 70 mL aqueous solution were added into the above mixture by maintaining pH 10 and continuous stirring for 1 h. After that, 10 mL of an aqueous solution containing 3.7 mmol FeCl_3_ and 1.8 mmol FeCl_2_ were slowly added into the cellulose medium over 15 min and maintaining pH 10. The nanocomposite was left over 24 h in the same medium for ageing and then filtered and washed with deionized water until neutral pH. Finally, the magnetic material was washed with ethanol and dried at 70 °C overnight and ground into a fine powder.

**Fe_3_O_4_@oleicacid nanocomposite synthesis**

The procedure proposed by Maaz et al., 2007 was used with slight modifications. to synthesize the oleic acid coated magnetic nanoparticles. The Fe_3_O_4_@oleicacid MNPs were generated dissolving 0.5g of dried Fe_3_O_4_ in 200 mL of 3M sodium hydroxide solution, before adding 2 mL of oleic acid, as surfactant and coating material. Then 25 mL of 18 mg mL^-1^ FeCl_3_·6H_2_O solution was added dropwise to the mixture while being stirred.The mixture was allowed to stand for 1 h at 80 °C to let the reaction efficiently proceed. The magnetic nanoparticles were washed twice with distilled water and then with ethanol to neutralize the basic medium and to remove the excess of oleic acid. Finally, the precipitate was dried at 70 °C overnight and ground into a fine powder.

**Fe_3_O_4_@Ag nanocomposite synthesis**

The magnetic Fe_3_O_4_@Ag nanocomposite material was synthesized following the procedure described by López-García et al. 2017 with slight modifications. For this, 20 mL of water were heated to approximately 80 °C and, while nitrogen gas was bubbled and the liquid continuously stirred, 0.56 g FeCl_3_·6H_2_O and 0.2 g FeCl_2_·4H_2_O were added. Once the solids were dissolved, a concentrated ammonia solution (2 mL) was incorporated, and the resulting suspension was stirred for 10 min. Next, the particles were separated by means of the magnet, and the supernatant was discarded. The solid remaining in the tube was washed several times until the washing liquids were neutral. The iron oxide particles thus obtained (approximately 0.28 g Fe_3_O_4_) were suspended in 20 mL of water and then covered with silver. For this, 5.7 mL of diluted silver nitrate solution (0.011 g L^−1^) were added, the mixture was stirred for 5 min and 14 mL of a diluted (0.012 g L^−1^) sodium tetrahydroborate solution were incorporated. The resulting mixture was further stirred for 10 min. The magnetic nanoparticles were washed twice with distilled water and then with ethanol. Finally, the precipitate was dried at 70 °C overnight and ground into a fine powder.

**Fe_3_O_4_@MWCNTs nanocomposite synthesis**

The magnetic Fe_3_O_4_@MWCNTs composite material was synthetized following the methodology described by Asgharinezhad and Ebrahimzadeh, 2015 .In a first step, MWCNTs were purified with 1 M nitric acid solution for 6 h at room temperature and then washed many times with distilled water and dried in an oven at 100 °C. Then, about 0.5 g of the purified nanotubes were added into 250 mL of a solution containing 0.85 g (NH_4_)_2_Fe(SO_4_)_2_⋅6H_2_O and 0.4222 g FeCl_3_ at 50 °C. After that, the suspension was sonicated for 20 min and 20 mL of 8 M ammonia solution was added dropwise to precipitate the magnetized particles while the solution was still under sonication. The pH was controlled to ensure that it remained in the 10–11 range by adding ammonia solution 25% (*w*/*w*). In order to enhance the whole growth of the nanoparticle crystals, the reaction was allowed to proceed at 50 °C for 30 min. The dispersion was cooled to room temperature, the Fe_3_O_4_@MWCNT were collected by a strong permanent magnet and washed three times with deionized water followed by ethanol. The magnetic composite substance was dried at 60 °C overnight and lastly ground in a mortar and kept at room temperature in an amber glass vial.

**Fe_3_O_4_@APTS nanocomposite synthesis**

The magnetic Fe_3_O_4_@APTS composite material was synthetized following the methodology described by Mukdasai et al. 2012. Firstly, magnetic nanoparticles were prepared by co-precipitation of aqueous solutions of FeCl_2_·4H_2_O (2.0 mg mL^-1^) and FeCl_3_ (2.4 mg mL^-1^). The mixture with volume ratio 2:1 of FeCl_3_:FeCl_2_·4H2O was homogenised with a homogeniser, rapidly heated to 160 °C and adjusted to neutral pH by addition of NH·OH, resulting in black iron oxide precipitates. Thereafter, the product was washed three times with water and dried in an oven at 90 °C. The surface of the magnetic nanoparticles was functionalised using the silylation reaction with some modifications. The dried magnetic nanoparticles (1.5 g), 3.0 g of 3-aminopropyl triethoxysilane (APTS) and 25 mL of 0.1% (v/v) acetic acid were stirred at room temperature for 30 min. The suspension was dried overnight in an oven at 80 °C. The obtained magnetic nanoparticles functionalised with 3-aminopropyl triethoxysilane (APTS magnetic nanoparticles) were washed with water and finally dried in an oven at 90 ºC.

**Fe_3_O_4_@MWCNTs/PPy nanocomposite synthesis**

The magnetic Fe_3_O_4_@MWCNTs/PPy composite material was synthetized following the methodology described by Asgharinezhad and Ebrahimzadeh, 2015. For this, 0.85 g of (NH_4_)_2_Fe(SO_4_)_2_·6H_2_O and 0.422 g of FeCl_3_·6H_2_O were dissolved in 250 mL of water, 0.5 g of carbon nanotubes (MWCNTs) were added to this solution and sonicated at 50ºC for 20 minutes. Then, 20 mL of an 8 M NH4OH solution was added dropwise to provoke, with this basic medium, the precipitation of the magnetic Fe_3_O_4_ nanoparticles on the MWCNTs walls, thus creating the magnetic nanomaterial. To complete its growth, the reaction was kept in a water bath at 50°C for 30 minutes. The MWCNTs/Fe_3_O_4_ precipitate was separated from the aqueous solution with the help of an external magnet, and the aqueous phase was then decanted. The nanomaterial was washed three times with pure water and ethanol. Finally, it was dried at 70°C overnight in an oven.

To obtain a more selective extraction material, it was functionalised by coating it with polypyrrole (PPy) chains, this was achieved by an oxidative polymerisation reaction on the already synthesised MWCNTs/Fe_3_O_4_ and washed with FeCl_3_ as oxidant. A mass of 0.6 g of MWCNTs/Fe_3_O_4_ was added to 250 mL of pure water at pH 9, adjusted with ammonia, and stirred for 5 min. 0.4 mL of pyrrole was then added, maintaining the stirring for another 10 min. Then, 0.8 g of sodium perchlorate was added and stirred for 5 min. Finally, a volume of 50 mL of an aqueous solution containing 0.56 g FeCl_3_ was added dropwise with constant stirring. The reaction was kept overnight at room temperature. Finally, the polypyrrole-functionalised magnetic nanomaterial was washed and dried.

**Supplementary References**

S36. Lett A, Sagadevan S, Alshahateet SF, et al (2021) Synthesis and characterization of polypyrrole-coated iron oxide nanoparticles. Mater Res Express 8:025007. https://doi.org/10.1088/2053-1591/abe253

S37. Luo YL, Fan LH, Xu F, et al (2010) Synthesis and characterization of Fe_3_O_4_/PPy/P(MAA-co-AAm) trilayered composite microspheres with electric, magnetic and pH response characteristics. Mater Chem Phys 120:590–597. https://doi.org/10.1016/j.matchemphys.2009.12.002

S38. Mukdasai S, Thomas C, Srijaranai S (2013) Analytical Methods Enhancement of sensitivity for the spectrophotometric determination of carbaryl using dispersive liquid microextraction combined with dispersive m-solid phase. Anal Methods 5:789–796. https://doi.org/10.1039/c2ay25838b

S39. Periyasamy S, Gopalakannan V, Viswanathan N (2017) Fabrication of magnetic particles imprinted cellulose based biocomposites for chromium (VI) removal. Carbohydr Polym 174:352–359. https://doi.org/10.1016/j.carbpol.2017.06.029

S40. López-García I, Rengevicova S, Muñoz-Sandoval MJ, Hernández-Córdoba M (2017) Speciation of very low amounts of antimony in waters using magnetic core-modified silver nanoparticles and electrothermal atomic absorption spectrometry. Talanta 162:309–315. https://doi.org/10.1016/j.talanta.2016.10.044

S41. Benedé JL, Chisvert A, Giokas DL, Salvador A (2014) Development of stir bar sorptive-dispersive microextraction mediated by magnetic nanoparticles and its analytical application to the determination of hydrophobic organic compounds in aqueous media. J Chromatogr A 1362:25–33. https://doi.org/10.1016/j.chroma.2014.08.024

S42. Alasl MRK, Sohrabi MR, Davallo M. (2019). Determination of trace amounts of aromatic amines after magnetic solid-phase extraction using silver-modified Fe_3_O_4_/graphene nanocomposite. J Sep Sci. 42:1777–85. https://doi.org/10.1002/jssc.201800896

S43. Mmelesi OK, Kuvarega A, Nkambule TTI, et al (2022) Synthesis of cobalt ferrite in one-pot-polyol method, characterization, and application to methylparaben photodegradation in the presence of peroxydisulfate. Mater Today Chem 26:101029. https://doi.org/10.1016/j.mtchem.2022.101029

S44. Houshiar M, Zebhi F, Razi ZJ, et al (2014) Synthesis of cobalt ferrite (CoFe_2_O_4_) nanoparticles using combustion, coprecipitation, and precipitation methods: A comparison study of size, structural, and magnetic properties. J Magn Magn Mater 371:43–48. https://doi.org/10.1016/j.jmmm.2014.06.059

S45. Nichea MJ, Palacios SA, Chiacchiera SM, et al (2015) Presence of multiple mycotoxins and other fungal metabolites in native grasses from a wetland ecosystem in Argentina intended for grazing cattle. Toxins 7:3309–3329. https://doi.org/10.3390/toxins7083309
